# Enduring modulation of dorsal raphe nuclei regulates (R,S)-ketamine-mediated resilient stress-coping behavior

**DOI:** 10.1038/s41380-024-02853-6

**Published:** 2024-11-26

**Authors:** Anderson Camargo, Anna Nilsson, Reza Shariatgorji, Ellen Appleton, Niclas Branzell, Daniel Doyon, Mattia Giovenzana, Xiaoqun Zhang, Daniel Dautan, Per E. Andren, Per Svenningsson

**Affiliations:** 1 Department of Clinical Neuroscience, Karolinska Institutet, Stockholm, Sweden; 2 Department of Pharmaceutical Biosciences, Spatial Mass Spectrometry, Science for Life Laboratory, Uppsala University, Uppsala, Sweden; 3 Department of Medicine and Surgery, University of Milano Bicocca Monza, Monza, Italy

**Keywords:** Neuroscience, Depression

## Abstract

Ketamine may be a novel pharmacologic approach to enhance resilience and protect against stress-related disorders, but the molecular targets underlying this response remain to be fully characterized. The multifunctional protein p11 is crucial in the pathophysiology of depression and antidepressant responses. However, it is still unclear whether p11 plays a role in the pro-resilience effects induced by ketamine. Here, we demonstrated that prophylactic administration of ketamine buffers passive stress-induced maladaptive phenotypes induced by chronic stress exposure. Spatial neurotransmitter and metabolite analysis revealed that prophylactic ketamine was also effective in blunting stress-induced disturbances of tryptophan metabolism in dorsal raphe nuclei (DRN). Additionally, we demonstrated that ketamine prevented chronic restraint stress-induced p11 reduction in DRN, a highly p11-enriched region. Furthermore, we provide novel evidence indicating that p11 deficiency regulates susceptibility to stress-induced depression-related phenotypes, and these behavioral maladaptations are dependent, at least in part, on p11 function in serotonergic neurons. Spatial neurotransmitter and metabolite analysis also showed a reduction of tryptophan and dopamine metabolism in DRN of serotonergic p11-deficient mice. Viral-mediated downregulation of p11 within DRN induced a stress-susceptible phenotype. Finally, our results also unveiled that the ability of ketamine to elicit a pro-resilience response against stress-induced maladaptive phenotypes was occluded when p11 was selectively deleted in serotonergic neurons. Altogether, we showed a previously unexplored role of the DRN circuit in regulating stress susceptibility and resilience-enhancing actions of ketamine.

## Introduction

Stress is an adaptive response regulated by the hypothalamic-pituitary-adrenocortical (HPA) axis that maintains homeostasis by triggering physiological changes under short-term adverse situations [1, 2]. While the acute stress response is an important and necessary mechanism to adapt to environmental changes, prolonged exposure to stress can cause molecular and cellular maladaptations that influence cognitive and emotional processing, ultimately contributing to the development of psychiatric disorders [3, 4]. For instance, chronic stress is the most common risk factor associated with major depressive disorder (MDD), one of the main causes of medical disability worldwide [5]. Although it has been shown that stress can underlie the development and relapse of MDD, it is important to note that not all stress-exposed individuals develop this psychiatric manifestation [6–8]. Therefore, uncovering what makes individuals stress-resilient or stress-susceptible could pave a new avenue for drug development not only to treat but also to prevent MDD [9].

Ketamine is a non-competitive N-methyl-D-aspartate (NMDA) receptor antagonist that has been used primarily as an anesthetic and commercially distributed in a racemic form composed of the enantiomers *R*-ketamine and *S*-ketamine [10]. However, the discovery of (*R,S*)-ketamine as a fast-acting antidepressant and its unique mechanism of action comprised one of the most substantial breakthroughs in MDD pharmacotherapy [11, 12]. These findings have led to the approval of the enantiomer *S*-ketamine nasal spray for the management of treatment-resistant MDD [13–15]. However, the use of *S*-ketamine presents some limitations, including potential drug adverse reactions such as dissociative symptoms, cognitive dysfunction and impaired working memory [16]. Although the rapid and sustainable actions of ketamine to treat MDD are remarkable [17, 18], innovative studies have also reported that (*R,S*)-ketamine may be a novel pharmacologic approach to enhance resilience and protect against stress-induced MDD in humans [19–22] and depressive-like state in rodents [23–26]. Nonetheless, the molecular targets and circuitries underlying ketamine’s pro-resilience response remain to be fully characterized [27, 28].

Numerous studies have started shedding light on the potential brain circuits and molecular targets underlying the resilience-enhancing effects of (*R,S*)-ketamine [28]. Noteworthy, a previous study demonstrated that persistent changes in dorsal raphe nuclei (DRN), the main brain region producing serotonin, mediates the pro-resilience effects elicited by (*R,S*)-ketamine [29]. However, the potential mechanisms underlying (*R,S*)-ketamine’s protective actions within DRN still remained to be fully uncovered. Within this scenario, p11 (also known as S100A10) has been identified as a key multifunctional protein underlying the pathophysiology of MDD and responses to antidepressant strategies [30]. The p11 levels are found to be reduced in brain tissue from patients diagnosed with MDD [31–33] and rodents subjected to chronic stress models that induce depressive-like behaviors [34, 35]. Notably, p11 deficiency elicits behavioral and molecular alterations that resemble clinical MDD [32, 33], including HPA axis hyperresponsiveness along with increased stress reactivity [36]. More importantly, this p11-mediated regulation of stress hyperactivity is dependent on serotonin transporter (SERT)-expressing neurons projecting from DRN [36].

Indeed, DRN is one of the brain areas with the highest expression of p11 transcripts [36, 37] and DRN-projecting neurons have been demonstrated to modulate responses to stressors and stress-coping behaviors [38–40]. Notably, a recent study showed that a reduction of p11 levels in DRN mediates depression-like behaviors [41]. Moreover, p11 has been reported to underlie the long-lasting, but not rapid, antidepressant-like effects of (*R,S*)-ketamine in mice [42], while our research group recently demonstrated that p11 is a potential predictor of (*R,S*)-ketamine response in patients with treatment-resistant MDD [43]. Given this scenario and involvement of DRN in (*R,S*)-ketamine’s pro-resilience effects, we hypothesize that p11 within DRN may be a potential target underlying its protective actions against stress. Therefore, the present study was designed to address this hypothesis using environmental stress and genetic p11 deficiency mouse models, behavioral testing, spatial neurotransmitters and metabolites mass spectrometry, and molecular analysis.

## Material and methods

### Animals

Wild-type (WT), constitutive global p11 knockout (p11KO), or p11 heterozygous (p11HET), p11 floxed WT (p11^*flx/flx*^), conditional knockout of p11 in Sert-expressing neurons (Sert-p11cKO, p11^*flx/flx*^ Sert–Cre^+/−^), or conditional knockout of p11 in choline acetyltransferase (ChAT)-expressing neurons (ChAT-p11cKO, p11^*flx/flx*^ ChAT–Cre^+/−^) mice were generated as previously described [36] on a C57BL/6J background. Genotypes were confirmed by PCR. All experiments were approved by the Karolinska Institutet Ethical Committee according to Swedish guidelines in full compliance with European requirements.

### Drugs

(*R,S*)-ketamine was purchased from Sigma-Aldrich (St. Louis, MO), dissolved in sterile saline (0.9% NaCl), and administered via intraperitoneal (i.p.) route at a dose of 15 mg/kg [44]. Ketamine was freshly prepared before administration and administered in a volume of 10 ml/kg body weight.

### Experimental design

Information regarding the experimental design, including behavioral testing, fluorescent in situ hybridization (RNA Scope), in situ hybridization, immunofluorescence, stereotaxic surgical procedures for viral injection, and matrix-assisted laser desorption/ionization-mass spectrometry imaging (MALDI-MSI) are included in the Supplementary Information.

### Statistical analysis

All statistical analyses were done using GraphPad Prism (GraphPad, San Diego, CA, USA). The D’Agostino–Pearson test was used to assess data normality. The differences among experimental groups were determined by two-tailed unpaired Student’s *t* test or two-way analysis of variance (ANOVA) followed by Tukey’s post hoc test, when appropriate. Differential abundance analysis of neurotransmitters and metabolites (shown in volcano plots) were performed using independent t-tests and the Benjamini–Hochberg multiple-testing correction to control the false discovery rate (FDR < 0.05). The details of statistical tests and their outcomes are presented in the Supplementary Information. Data are presented as mean ± standard error of the mean (SEM). A value of *P* < 0.05 was considered significant.

## Results

### Ketamine prophylactic administration buffers passive stress-coping behaviors induced by chronic stress exposure

**Fig. 1:**
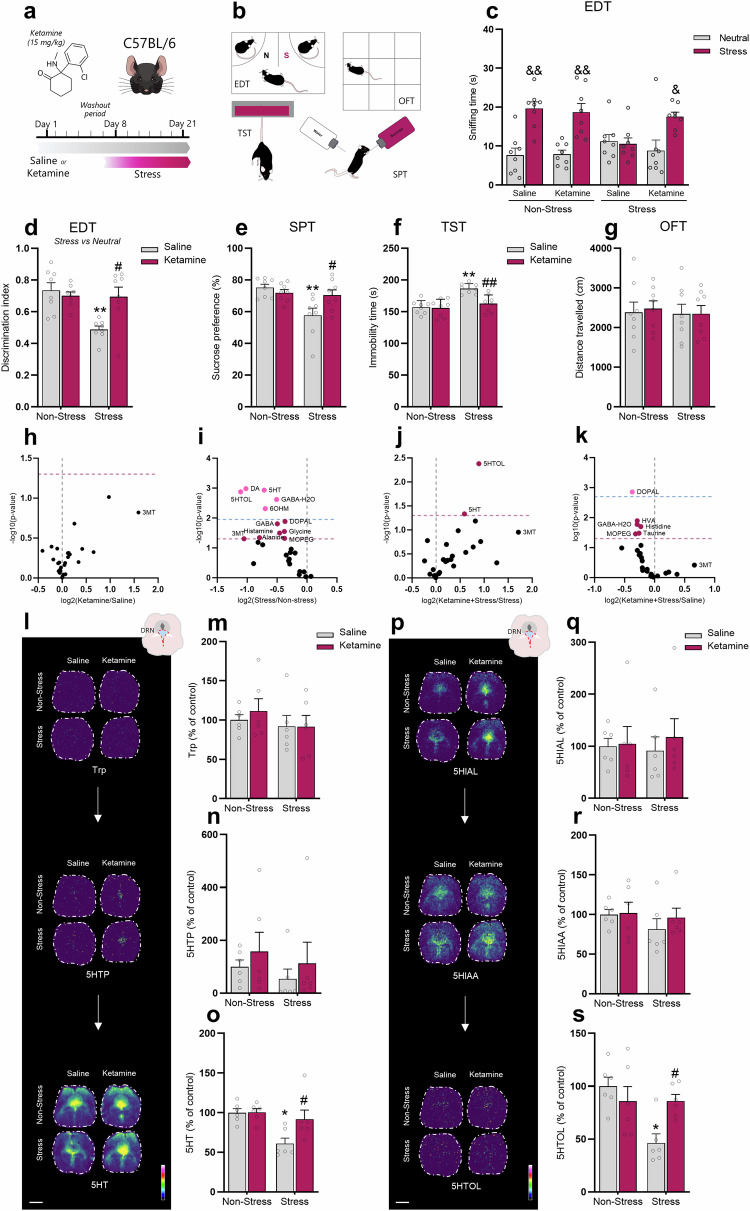
Prophylactic treatment of (*R-S*)-ketamine buffers passive stress-coping behaviors and alterations in tryptophan metabolism in DRN induced by chronic stress exposure. **a** Experimental timeline of the treatment protocol, where WT mice received a single saline or ketamine administration (15 mg/kg, racemic mixture), followed by a 1-week washout period before the exposure to chronic restraint stress protocol. **b** Schematic illustration of behavioral tests. **c** Ketamine effectively prevented stress-induced reduced time sniffing the stressed stimulus in the EDT. **d** Ketamine was effective in blunting stress-induced impairment in the emotion discrimination index. **e** Ketamine effectively prevented stress-induced anhedonic-like behavior in the SPT. **f** Ketamine buffered passive coping behaviors in the TST induced by stress exposure. **g** There were no significant differences observed in distance traveled in any experimental group in the OFT. **h** Volcano plots depicting the distribution of neurotransmitters and metabolites fold differences and -log*10* (non-adjusted *p*-value and FDR-adjusted *p*-value) in DRN of ketamine-treated mice compared to saline-administered mice. **i** Volcano plots showing the indicated fold differences and -log*10* (non-adjusted *p*-value and FDR-adjusted *p*-value) of all detected neurotransmitters and metabolites in DRN of stressed mice compared to non-stressed mice. **j** Volcano plots showing the indicated fold differences and -log*10* (non-adjusted *p*-value and FDR-adjusted *p*-value) of all detected neurotransmitters and metabolites in DRN of mice treated prophylactically with ketamine and exposed to stress compared to stressed mice administered with saline. **k** Volcano plots demonstrating the distribution of neurotransmitters and metabolites fold differences and -log*10* (non-adjusted *p*-value and FDR-adjusted *p*-value) in DRN of ketamine-treated mice exposed to stress compared to saline-treated mice under non-stress condition. **l**–**s** Ion images and bar graphs showing the quantification of the tryptophan and metabolite peaks in DRN of non-stressed or stressed mice previously treated with saline or ketamine. Values are expressed as means ± S.E.M (*n* = 6). Individual data are represented as dots. &*p* < 0.05, &&*p* < 0.01 com*p*ared with the neutral stimulus; *p < 0.05, **p < 0.01 compared with the non-stressed saline-treated group; #*p* < 0.05, ##*p* < 0.01 compared with stressed saline-treated group (two-way ANOVA followed by Tukey’s post hoc test). 5HTP 5-hydroxytryptophan, 5HT serotonin, 5HIAL 5-hydroxyindoleacetaldehyde, 5HIAA 5-hydroxyindoleacetic acid, 5-HTOL 5-hydroxytryptophol, 6OHM -6-hydroxymelatonin, Trp tryptophan. Images were acquired at a lateral resolution of 1 mm. Data are shown using a rainbow scale (0–100%).

Exposure to prolonged stress is one of the main risk factors underlying the development of MDD [45], and the chronic restraint stress protocol has provided an especially useful model for exploring and mimicking the molecular and physiological adaptations that occur in clinical MDD [34]. In contrast, ketamine has been reported as a promising pro-resilience agent against stress-induced development of maladaptive behaviors [23, 24]. For this reason, we tested (*R,S*)-ketamine administration as a pro-resilience strategy. To address this experimental approach, WT mice received a single i.p. administration of saline or (*R,S*)-ketamine (ketamine, 15 mg/kg) and were subjected to a 1-week washout period before the exposure to stress protocol (Fig. 1a). Next, animals were subjected to the chronic restraint stress procedure (2 h/day, for 14 days, using a 50 ml falcon tube) and then underwent behavioral testing, which included the emotion discrimination test (EDT, a marker of emotion information-related cognitive control) [46], the tail suspension test (TST, a measure of behavioral despair) [47], the sucrose preference test (SPT, a measure of reward or pleasure behavior) [48], and the open-field test (OFT, a marker of locomotor activity) [36] (Fig. 1b).

Our results show that the saline- or ketamine-treated WT mice spent more time sniffing the acutely stressed stimulus when the discrimination task was between stress and neutral conspecifics (*p* < 0.01, Fig. 1c). Also, the chronic stress protocol elicited emotion discrimination impairment (*p* < 0.01), but this behavioral maladaptation was buffered by ketamine prophylactic administration (*p* < 0.05, Fig. 1c). The administration of ketamine 1 week before the onset of the restraint stress protocol effectively prevented stress-induced reduction in the discrimination index (*p* < 0.05, Fig. 1d). No significant bias was detected in the EDT using a neutral *versus* neutral condition (Supplementary Fig. 1a, b). Chronic restraint stress also elicited a depressive-like behavior in the SPT (*p* < 0.05, Fig. 1e) and TST (*p* < 0.05, Fig. 1f), as evidenced by the reduced sucrose preference and increased immobility, respectively. However, ketamine, when administered 1 week before the onset of the restraint stress protocol, was effective in blunting the passive stress-coping behaviors induced by this stressor stimulus in both behavioral tests (*p* < 0.05, Fig. 1e, f). No effects were detected in the OFT (Fig. 1g).

### Ketamine selectively blunts stress-induced disturbances on tryptophan metabolism in DRN

Considering the involvement of DRN in the pro-resilience effects displayed by ketamine prophylactic administration against stress [29], we sought to investigate how neurotransmitters and their metabolites were affected in the DRN. We conducted MALDI-MSI to spatially characterize neurotransmitters and metabolites changes (Fig. 1h–s, Supplementary Fig. 2). Differential abundance analysis of all identified metabolites across the measured groups revealed alterations in DRN (Fig. 1h–k). We also performed two-way ANOVA to examine the changes in the DRN. We found a main effect of stress, with stress-exposed mice presenting a significant reduction of 6OHM, DA, DOPAL, HVA, MOPEG, GABA, glycine, alanine, and adenine in DRN, regardless the administration of saline or ketamine (*p* < 0.05, Supplementary Fig. 2a). A main effect of ketamine was also observed, with ketamine-treated mice presenting a significant increase on 3MT and EP levels in DRN, regardless of exposure to stress (*p* < 0.05, Supplementary Fig. 2a). We also found a significant interaction for ketamine x stress in the levels of 5HT and 5HTOL, in which stress caused a significant reduction of 5HT (Fig. 1l, o) and 5HTOL (Fig. 1p,s) levels in DRN (*p* < 0.05), but these alterations were not observed in mice prophylactically treated with ketamine (*p* < 0.05). No significant effects were observed in the levels of tryptophan (Fig. 1l, m), and 5HT metabolites, e.g., 5HTP (Fig. 1l, n), 5HIAL (Fig. 1p, q), and 5HIAL (Fig. 1p, r). These findings suggest that the ability of ketamine in buffering stress-induced alterations on 5HT and its metabolite 5HTOL might be associated with the resilient stress-coping phenotype.

### p11 levels in DRN are modulated by stress and ketamine prophylactic administration

**Fig. 2:**
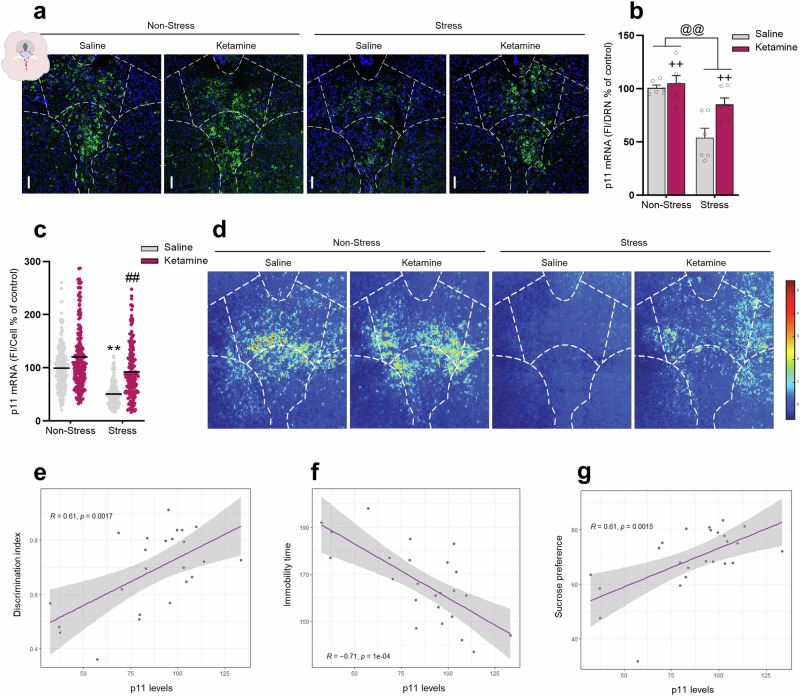
p11 levels in DRN are modulated by chronic stress exposure and prophylactic treatment of (*R-S*)-ketamine. **a** Representative fluorescent in situ hybridization images showing p11 (green) mRNA levels and DAPI (blue) staining in the DRN from saline-treated or ketamine-treated mice subjected or not to chronic stress protocol. **b** Chronic stress-induced p11 downregulation within DRN is alleviated by prophylactic ketamine administration. **c** Quantification of fluorescence in individual p11-containing cells in the DRN (*n* = 200 p11-positive cells from 6 mice). **d** Colored raster plot of the z-score of p11 mRNA levels in the DRN across all saline-treated or ketamine-treated mice subjected or not to chronic stress protocol. Pearson´s correlation coefficient between p11 levels and (**e**) discrimination index in the emotion discrimination test, (**f**) immobility time in the tail suspension test, and (**g**) sucrose preference in the sucrose preference test. Values are expressed as means ± S.E.M (*n* = 6–8). Individual data are represented as dots. **p < 0.01 compared with the non-stressed saline-treated group; ##*p* < 0.01 compared with stressed saline-treated group (two-way ANOVA followed by Tukey’s post hoc test). @@ *p* < 0.01 compared with non-stressed mice (i.e., a significant main effect of stress protocol); ++*p* < 0.01 compared with saline-treated mice subjected or not to stress protocol (i.e., a significant main effect of ketamine treatment).

The p11 is a key multifunctional protein underlying stress response, MDD onset, and antidepressant effects [30, 36]. Interestingly, DRN is one of the brain areas with the highest expression of p11 transcripts [36, 37] and a recent study showed that a reduction of p11 levels in DRN mediates depression-like behaviors [41]. Next, we evaluated whether p11 expression in DRN was changed by ketamine prophylactic administration (Fig. 2). Our results showed that ketamine administration per se did not change DRN p11 expression in non-stressed mice. In addition, mice exposed to chronic restraint stress exhibited significantly reduced DRN p11 expression compared to the non-stressed control group (*p* < 0.01, Fig. 2a, b), but this alteration was alleviated by ketamine when prophylactically administrated 1 week before the onset of stress stimulus (*p* < 0.05, Fig. 2a, b). In addition, we also observed that the reduced p11 mRNA expression in individual DRN neurons induced by stress was blunted by ketamine (*p* < 0.01, Fig. 2c, d). A significant correlation coefficient between p11 levels and ketamine/stress effects in the EDT (Fig. 2e), SPT (Fig. 2f), and TST (Fig. 2g) was detected. This data suggests that the resilient phenotypes induced by ketamine prophylactic administration might be associated with persistent changes in p11 expression within the DRN circuit.

### p11 deficiency regulates susceptibility to stress-induced depression-related phenotypes

**Fig. 3:**
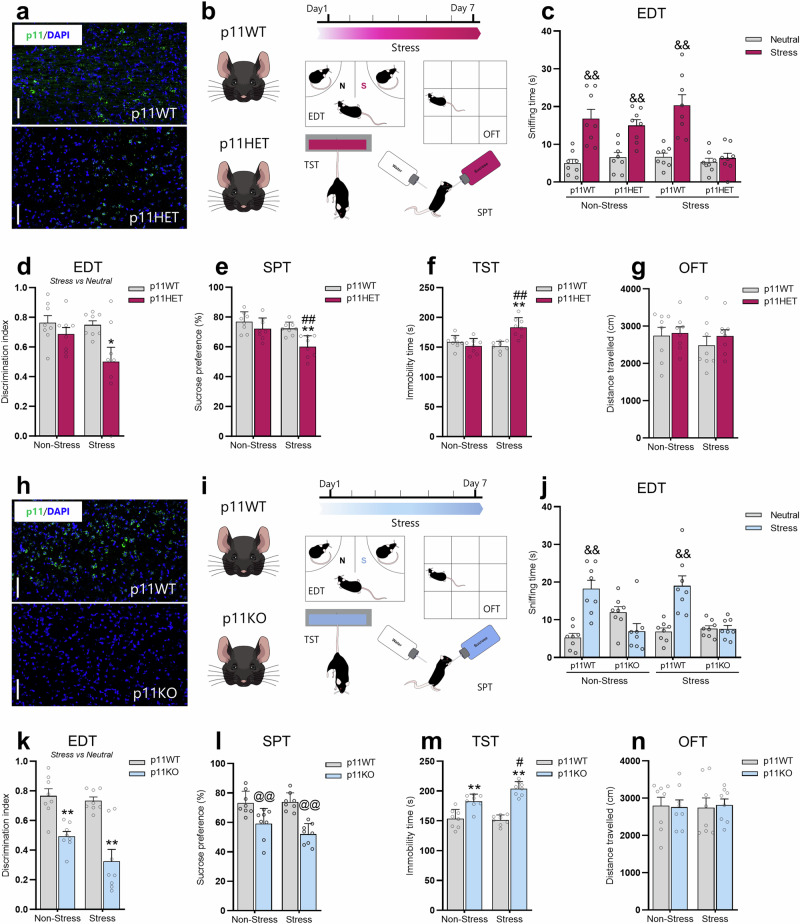
p11 deficiency regulates susceptibility to stress-induced depression-related phenotypes. **a** Representative fluorescent in situ hybridization (RNA scope) in the DRN showing p11 (green) and DAPI (blue) staining in p11WT or p11HET. Scale bars: 100 µm. **b** Timeline of the experimental protocol and behavioral tests. **c** p11HET mice displayed reduced time sniffing the acutely stressed stimulus as compared to the neutral conspecific in the emotion discrimination test (EDT) following the subthreshold stress procedure. **d** Subthreshold stress exposed-p11HET mice exhibited emotion discrimination impairment. **e** p11HET mice presented a susceptible phenotype in the sucrose preference test (SPT) following the subeffective stress challenge. **f** p11HET mice showed passive coping behavior in the tail suspension test (TST) after subthreshold stress stimulus. **g** All experimental groups presented a comparable distance traveled in the open-field test (OFT). Values are expressed as means ± S.E.M (*n* = 8). &&*p* < 0.01 compared with the neutral stimulus; *p < 0.05, **p < 0.01 compared with the non-stressed p11WT group; ##*p* < 0.01 compared with the non-stressed p11HET group (two-way ANOVA followed by Tukey’s post hoc test). **h** RNA scope image depicting p11 mRNA levels (green) and DAPI (blue) in DRN from p11WT and p11KO mice. Scale bars: 100 µm. **i** Representative timetable of the experimental protocol and behavioral tests. **j** p11KO mice presented a reduction in the time sniffing the stressed conspecific as compared to the neutral one in the EDT. **k** p11KO mice showed emotion discrimination impairment regardless of stress challenge. **l** p11KO mice presented an anhedonic behavior in the SPT. **m** p11KO mice displayed behavioral despair in TST. **n** All groups exhibited a comparable distance traveled in the OFT. Values are expressed as means ± S.E.M (*n* = 8). Individual data are represented as dots. &&*p* < 0.01 compared with the neutral stimulus; **p < 0.01 compared with the non-stressed p11WT group; #*p* < 0.05 compared with the non-stressed p11KO group (two-way ANOVA followed by Tukey’s post hoc test). @@*p* < 0.01 compared with mice subjected or not to stress procedure (i.e., a significant main effect of genotype).

Considering that the stress protocol induces p11 reduction associated with depression-like phenotype, we next investigated whether genetic p11 downregulation would be sufficient to make mice more prone to stress-induced maladaptive behaviors. To address this hypothesis, p11HET mice were used to address this hypothesis, as they are characterized by a partial global reduction of p11 (Fig. 3a). These mice were exposed to the subthreshold restraint stress protocol (2 h/day, for 7 days, using a 50 ml falcon tube), which has been reported to be a submaximal restraint stress procedure insufficient for producing a susceptible phenotype in naïve mice [49]. On the testing day, 24 h after the last stress episode, mice were tested in the EDT, SPT, TST, and OFT (Fig. 3b). We observed that the p11WT and p11HET non-stressed mice spent more time sniffing the acute stressed stimulus when the discrimination task was between a stress and a neutral conspecific (*p* < 0.01, Fig. 3c). However, we detected that after the subthreshold stress, while p11WT presented the same behavioral response (*p* < 0.01), p11HET mice displayed impaired emotion discrimination (Fig. 3c). In the discrimination index (Fig. 3d), we found that p11HET group subjected to the subthreshold stress displayed a significant reduction when compared to the non-stressed p11WT group (*p* < 0.05). No significant differences were observed in the EDT when the discrimination task was between neutral *versus* neutral stimulus (Supplementary Figure 3a, b). Additionally, we also detected that subthreshold stress-exposed p11HET mice exhibited significantly decreased sucrose preference in the SPT (*p* < 0.01, Fig. 3e) and increased immobility in the TST (*p* < 0.01, Fig. 3f) when compared to the non-stressed p11WT and p11HET groups. No alteration was observed in the distance traveled in the OFT in any experimental group (Fig. 3g). These findings suggest that a global downregulation of p11 contributes to the susceptibility to stress-induced depression-like states.

To further determine the role of p11 reduction in regulating stress-susceptible phenotypes, we investigated the impact of p11 knockout against stress-induced maladaptive behaviors. Thus, we tested p11KO mice (Fig. 3h) using the same design of subthreshold stress procedure and behavioral testing as above (Fig. 3i). We detected that p11WT, but not p11KO, spent more time sniffing the acutely stressed stimulus when compared to the neutral stimulus (*p* < 0.01) in the EDT, and this behavioral pattern was also observed after the subeffective stressor (*p* < 0.01, Fig. 3j). Moreover, p11KO displayed a significant reduction in the discrimination index when compared to the p11WT (*p* < 0.01; Fig. 3k) regardless of stress challenge. No significant differences were observed in the EDT when the discrimination task was between neutral *versus* neutral stimulus (Supplementary Fig. 3c, d). Furthermore, our results showed that p11KO mice presented a significant reduction in sucrose preference in the SPT (*p* < 0.01, Fig. 3l) and an increase in immobility in the TST (*p* < 0.01, Fig. 3m), while no alteration was detected in the distance traveled in the OFT (Fig. 3n). Interestingly, p11KO mice exposed to stress presented a more pronounced depression-like phenotype in the TST (*p* < 0.05, Fig. 3m). Therefore, our findings demonstrate that p11 deficiency makes mice more prone to stress-induced maladaptive behaviors.

### p11 deficiency in Sert-, but not in ChAT-, expressing neurons mediates susceptibility to stress-induced depression-related phenotypes

**Fig. 4:**
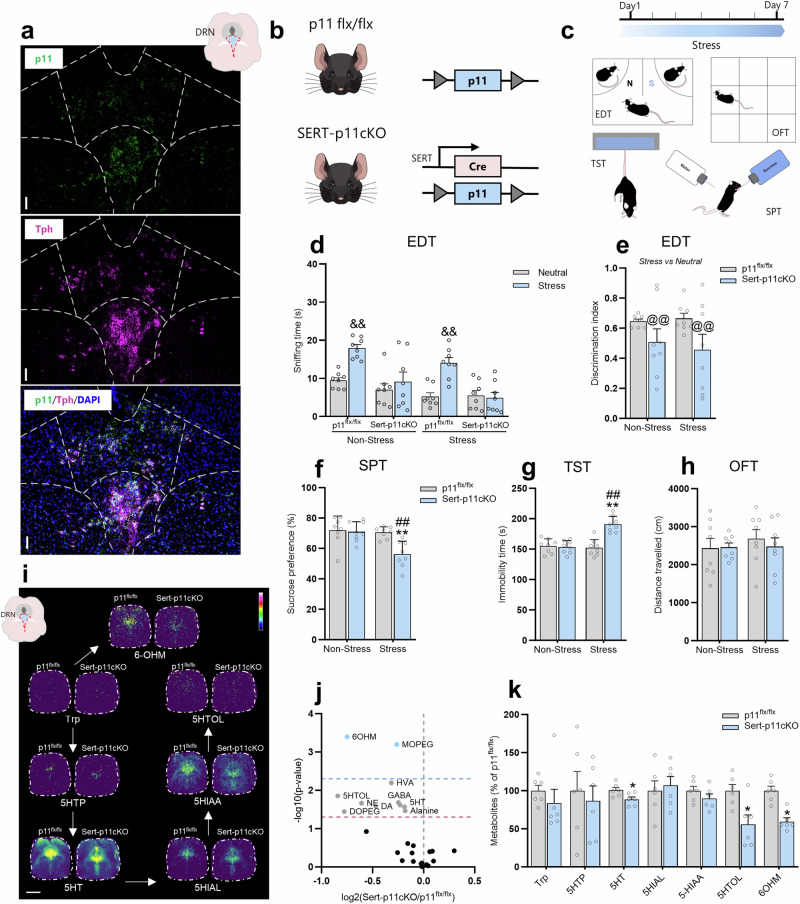
p11 loss in Sert-expressing neurons mediates susceptibility to stress-induced depression-related phenotypes. **a** Representative RNA scope image illustrating that p11 transcripts (green) are strongly expressed Tph-positive cells (magenta) in the DRN. DAPI (blue) staining. Scale bars: 100 µm. **b** Schematic depiction of transgenic Sert-p11cKO (p11^*flx/flx*^ SERT–Cre^+/−^) mouse construct. **c** Experimental time plan and behavioral tests. **d** Sert-p11cKO mice showed reduced time sniffing when comparing the stressed stimulus to the neutral conspecific in the EDT. **e** Sert-p11cKO mice displayed emotion discrimination index impairment. **f** Sert-p11cKO elicited a susceptible phenotype in the SPT following subeffective stress stimulus. **g** Sert-p11cKO mice exhibited passive coping behavior in the TST after the subthreshold stress challenge. **h** All groups presented comparable locomotor activity in the OFT. Values are expressed as means ± S.E.M (n = 8). Individual data are represented as dots. &&*p* < 0.01 compared with the neutral stimulus; **p < 0.01 compared with the non-stressed p11^*flx/flx*^ group; ##*p* < 0.01 compared with the non-stressed Sert-p11cKO group (two-way ANOVA followed by Tukey’s post hoc test). @@*p* < 0.01 compared with p11^*flx/flx*^ mice subjected or not to stress procedure (i.e., a significant main effect of genotype). **i** Ion images of tryptophan and metabolite peaks in DRN from p11^*flx/flx*^ and Sert-p11cKO. **j** Volcano plots showing the indicated fold differences and -log*10* (non-adjusted *p* value and FDR-adjusted *p*-value) of all detected neurotransmitters and metabolites in DRN of Sert-p11cKO mice compared to p11^*flx/flx*^ mice. **k** Bar graph showing the quantification of the tryptophan and metabolite peaks. Values are expressed as means ± S.E.M (*n* = 6). Individual data are represented as dots. *p < 0.05 compared with the non-stressed p11^*flx/flx*^ group (Student’s *t* test). 5HTP 5-hydroxytryptophan, 5HT serotonin, 5HIAL 5-hydroxyindoleacetaldehyde, 5HIAA 5-hydroxyindoleacetic acid, 5-HTOL 5-hydroxytryptophol, 6OHM -6-hydroxymelatonin, Trp tryptophan. Images were acquired at a lateral resolution of 1 mm. Data are shown using a rainbow scale (0–100%).

p11 has been reported to be enriched in distinct neuronal types, such as mossy and basket cells in the dentate gyrus of the hippocampal formation [50], layer 5 corticostriatal neurons [51], cholinergic neurons in the nucleus accumbens [52] and laterodorsal tegmentum [37], as well as serotonergic neurons (Fig. 4a) in the DRN [33, 36, 53]. Therefore, we next sought to determine which neuronal subtypes are involved in p11 deficiency-induced stress-susceptibility to depression-like behaviors. To evaluate this, we first deleted p11 specifically in Sert-expressing neurons (Supplementary Fig. 4a, b). This was achieved using a conditional knockout strategy by crossing p11^*flx/flx*^ mice with Sert-Cre reporter mice, which enables the investigation of p11 within the serotonergic system in stress-relevant behaviors. We, therefore, tested the conditional knockout of p11 in Sert-expressing neurons (Sert-p11cKO, p11^*flx/flx*^ SERT–Cre^+/−^) mice using the same experimental design of subthreshold stress and behavioral testing described before (Fig. 4a–c). Our results show that the p11^*flx/flx*^, but not Sert-p11cKO, mice spent more time sniffing the acutely stressed stimulus when the discrimination task was between stress and neutral conspecifics (*p* < 0.01, Fig. 4d), regardless of stress procedure. We also found that stress-exposed Sert-p11cKO mice demonstrated a significant reduction in the discrimination index (Fig. 4e), regardless of stress protocol (*p* < 0.01). No significant alterations were detected in the EDT when the discrimination task was between neutral *versus* neutral stimulus in any experimental group (Supplementary Figure 4c, d). Moreover, subthreshold stress-exposed Sert-p11cKO mice also exhibited significantly decreased sucrose preference in the SPT (*p* < 0.01, Fig. 4f) and increased immobility in the TST (*p* < 0.01, Fig. 4g) as compared to the non-stressed p11^*flx/flx*^ and Sert-p11cKO mice (*p* < 0.01). All groups had comparable distance traveled in the OFT (Fig. 4h). These results suggest that p11 deficiency in Sert-expressing neurons might contribute to the susceptibility to stress-induced depression-like phenotypes in a task-dependent manner.

Since Sert-p11cKO mice showed susceptibility to stress, we sought to investigate how neurotransmitters and their metabolites were affected in the DRN. We also used MALDI-MSI to spatially characterize neurotransmitters and metabolites in DRN of stress-exposed and ketamine-treated mice (Fig. 4i, Supplementary Fig. 5). Differential abundance analysis of all identified neurotransmitters and their metabolites revealed alterations in the DRN of Sert-p11cKO mice (Fig. 4j). For example, Sert-p11cKO mice displayed a significant reduction of metabolites related to tryptophan (5HT, 5HTOL, and 6OHM, *p* < 0.05, Fig. 4k). Moreover, the results also showed that Sert-p11cKO mice exhibited altered levels of DA, HVA, NE, DOPEG, MOPEG, GABA, and alanine in DRN when compared to p11^*flx/flx*^ mice (*p* < 0.05, Supplementary Fig. 5a–c). This data suggests that p11 deficiency in SERT-expressing neurons induces disturbances in tryptophan and dopamine metabolism in DRN, which could be associated with the stress-susceptible phenotype.

To determine whether this regulation of stress-coping behaviors elicited by p11 is Sert-expressing neurons-specific, we decided to assess whether cholinergic cells expressing p11 (Supplementary Fig. 6a) can regulate stress-susceptibility, since this circuit was also found to play a role in p11 deficiency-induced depression-like states [52]. We, therefore, examined the impact of p11 condition knockout in ChAT-expressing neurons, using ChAT-p11cKO mice subjected to the same experimental design described before (Supplementary Fig. 6a–c). Results show that p11^*flx/flx*^ mice, but not ChAT-p11cKO, have the ability to distinguish between emotion stimulus, as evidenced by increased time sniffing the stressed conspecific when compared to the neutral one (*p* < 0.01) in the EDT, and this behavioral phenotype was also found after the subthreshold stressor (*p* < 0.01, Supplementary Fig. 6d). Similarly, ChAT-p11cKO displayed a significant reduction in the discrimination index when compared to the p11WT (*p* < 0.01; Supplementary Fig. 6e) regardless of stress stimulus. No significant bias was observed in the EDT in a neutral *versus* neutral condition (Supplementary Fig. 6f, g). Our data also indicated that ChAT-p11cKO mice presented a basal depression-like behavior, as observed by the decreased sucrose preference in the SPT (*p* < 0.01, Supplementary Fig. 6h) and increased immobility in the TST (*p* < 0.01, Supplementary Fig. 6i) when compared to the p11^*flx/flx*^ mice, regardless of stress protocol. No alteration was detected in the distance traveled in the OFT (Supplementary Fig. 6j). These findings suggest that the loss of p11 in ChAT-expressing neurons regulates basal depression-like phenotype, recapitulating global p11KO mice.

### Viral-mediated downregulation of p11 within DRN mediates stress-susceptible phenotype

**Fig. 5:**
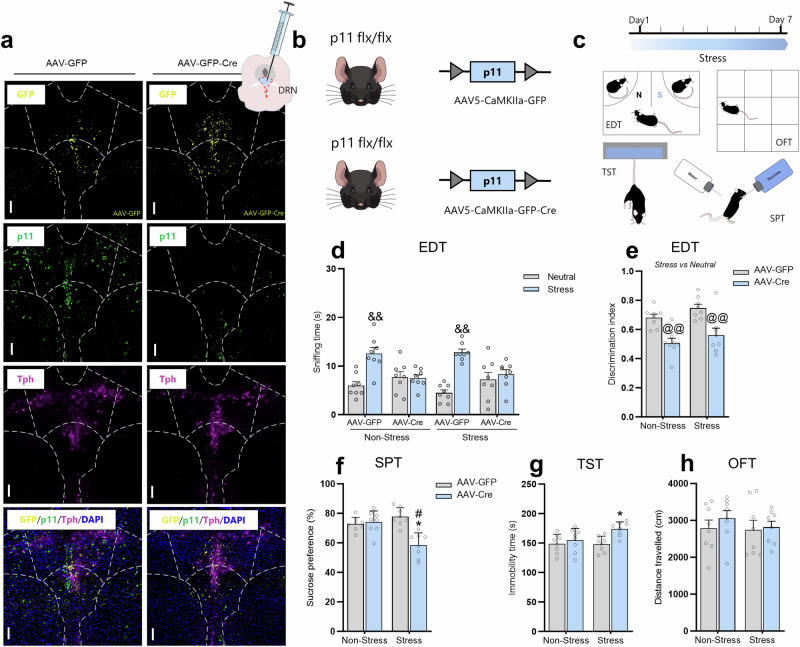
p11 within the DRN circuit mediates stress-susceptible phenotype. **a** Representative image of AAV-GFP and AAV-GFP-Cre virus expression (yellow), p11 levels (green), Tph-positive cells (magenta), and DAPI (blue) staining in the DRN from p11^*flx/flx*^ mice. Scale bars: 100 µm. **b** Schematic illustration of transgenic p11^*flx/flx*^ mouse injected with AAV-GFP or AAV-Cre viral construct. **c** Representative experimental time plan and behavioral tests. **d** Knockdown of p11 within DRN induced a reduction in the time sniffing the stressed stimulus as compared to the neutral one in the EDT. **e** AAV-Cre-injected mice showed emotion discrimination index impairment. **f** Knockdown of p11 within DRN makes mice more prone to maladaptive behavior in the SPT. **g** AAV-Cre-mediated p11 knockdown within the DRN group exhibited behavioral despair in TST after a subthreshold stress paradigm. **h** All groups exhibited a comparable distance traveled in the OFT. Values are expressed as means ± S.E.M (n = 8). Individual data are represented as dots. &&*p* < 0.01 compared with the neutral stimulus; *p < 0.05 compared with the non-stressed p11^*flx/flx*^ + AAV-GFP group; #*p* < 0.05 compared with the non-stressed p11^*flx/flx*^ + AAV-Cre group (two-way ANOVA followed by Tukey’s post hoc test). @@*p* < 0.01 compared with p11^*flx/flx*^ + AAV-GFP mice subjected or not to stress procedure (i.e., a significant main effect of genotype).

To further assess the role of the serotonin system in p11-induced susceptibility to stress-induced depression-like phenotypes, viral-mediated functional experiments were performed to manipulate the expression of p11 within DRN. It is important to note that other regions, such as the median raphe nucleus (MRN), also project to the brain and might be important in serotonergic circuitry. However, DRN is the main brain region producing serotonin. For this reason, this experiment was designed to focus on the role of DRN in p11-induced stress susceptibility. We determined the effects of p11 downregulation, injecting AAV5-CaMKIIa-GFP (AAV-GFP, control virus) or AAV5-CaMKIIa-GFP-Cre (AAV-Cre) into DRN of p11^*flx/flx*^ (Fig. 5a). Next, AAV-injected p11^*flx/flx*^ mice underwent subthreshold stress procedure and behavioral testing (Fig. 5b, c). We observed that AAV-Cre injection into DRN significantly reduced p11 levels in this brain region, but not in MRN (Supplementary Fig. 7a–c). p11 downregulation induced by AAV-Cre effectively impaired emotion discrimination in both non-stressed and stressed mice subjected to the EDT (*p* < 0.01, Fig. 5d). This behavioral phenotype was further confirmed by calculating the discrimination index (*p* < 0.01, Fig. 5e). No significant bias was detected in the EDT using a neutral *versus* neutral condition (Supplementary Fig. 7d,e). We also found that stress-exposed AAV-Cre-, but not AAV-GFP-, injected mice presented a significant reduction in the sucrose preference in the SPT (*p* < 0.05, Fig. 5f) and an increase in the immobility time in the TST (*p* < 0.05, Fig. 5g) as compared to the non-stressed AAV-GFP-injected mice (*p* < 0.05). No significant differences were observed in the OFT (Fig. 5h). These findings indicate that viral-mediated downregulation of p11 within DRN recapitulates the passive stress-coping behaviors observed in Sert-p11cKO. This further reinforces the role of p11 deficiency in Sert-expressing neurons in regulating susceptibility to stress-induced depression-like states.

### p11 in serotonergic neurons underlie ketamine-induced resilient stress-coping behaviors

**Fig. 6:**
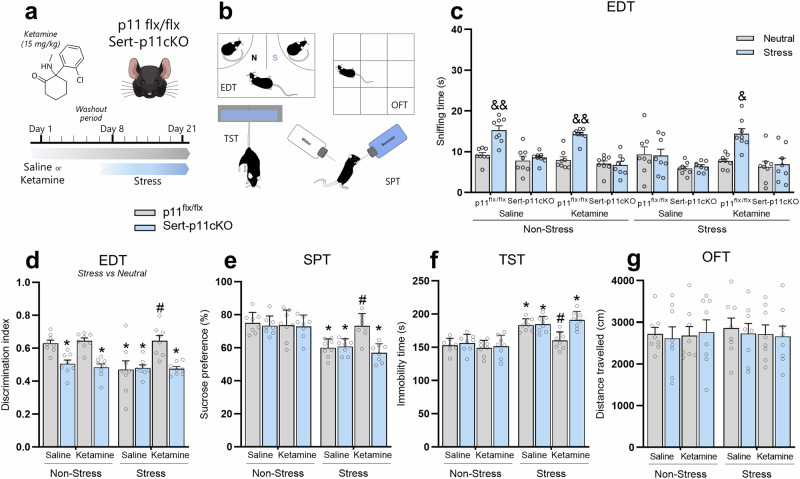
The resilient phenotype induced by prophylactic ketamine administration is dependent on p11 expression in serotonergic neurons. **a** Schematic timeline of the treatment protocol, where p11^*flx/flx*^ or Sert-p11CKO mice received a single administration of saline or ketamine (15 mg/kg, racemic mixture), followed by a 1-week washout period before the exposure to chronic restraint stress protocol. **b** Experimental protocol of behavioral tests. **c** Effects of ketamine prophylactic administration in the time sniffing stress *versus* neutral stimuli in the EDT. **d** Ketamine’s ability to buffer stress-induced emotion discrimination impairment is abolished in Sert-p11cKO. **e** Resilient phenotype induced by prophylactic ketamine against stress-induced anhedonic-like behavior in the SPT is dependent on p11 expression in Sert-expressing neurons. **f** Prophylactic efficacy elicited by ketamine against stress-induced passive coping behavior in the TST is occluded by p11 deficiency in Sert-expressing neurons. **g** There were no significant differences observed in distance traveled in any experimental group in the OFT. &*p* < 0.05, &&*p* < 0.01 com*p*ared with the neutral stimulus; *p < 0.05 compared with the non-stressed saline-treated p11^*flx/flx*^ group; #*p* < 0.05 compared with stressed saline-treated p11^*flx/flx*^ group (two-way ANOVA followed by Tukey’s post hoc test).

In line with the notion that the pro-resilience properties of ketamine may involve p11 modulation within DRN, we next sought to identify whether the p11-related serotonergic circuit underlies ketamine-induced resilient stress-coping phenotype. p11^*flx/flx*^ or Sert-p11cKO mice received a single i.p. administration of saline or ketamine (15 mg/kg) and were subjected to a 1-week washout period prior to the exposure to stress for 14 days (Fig. 6a). Next, animals were then tested in the behavioral paradigms (Fig. 6b). We found that p11^*flx/flx*^, but not Sert-p11cKO, mice significantly spent more time sniffing the stressed stimulus compared to a neutral conspecific (*p* < 0.01, Fig. 6c), regardless of the administration of saline or ketamine. Chronically stress-exposed p11^*flx/flx*^ mice presented impairment in emotion discrimination, but this behavioral alteration was prevented by ketamine (*p* < 0.01, Fig. 6c). However, this ketamine-induced resilient phenotype was not detected in Sert-p11cKO mice. Using the discrimination index, we found that Sert-p11cKO mice have a significant reduction in this task, regardless of the saline or ketamine treatment (*p* < 0.05, Fig. 6d). We also observed that while ketamine prevented chronic stress-induced discrimination index reduction in p11^*flx/flx*^ mice (*p* < 0.05, Fig. 6d), it failed to present the same response in Sert-p11cKO. No significant differences were detected in the EDT using neutral *versus* neutral conspecifics (Supplementary Fig. 8a, b). Interestingly, ketamine significantly buffered stress-induced decrease in the sucrose preference in the SPT (*p* < 0.05, Fig. 6e) and increase in the immobility in the TST (*p* < 0.05, Fig. 6f) in p11^*flx/flx*^ mice, but the effect was completely occluded in Sert-p11cKO mice. No significant differences were observed in the OFT (Fig. 6g). These findings suggest that p11 within the serotonergic circuit might be a key protein underlying the ability of ketamine to elicit a resilient stress-coping phenotype.

## Discussion

The present study reinforces and extends the notion that ketamine when given prophylactically is effective in buffering stress-induced maladaptive behaviors. We now expand these findings indicating that enduring modulation of dorsal raphe nuclei is involved in the ability of (R,S)-ketamine to mediate resilient stress-coping behavior. Additionally, we provide novel evidence demonstrating that p11 deficiency regulates susceptibility to stress-induced depression-related phenotypes, and these behavioral maladaptations are dependent, at least in part, on p11 function in serotonergic neurons. Finally, this study identifies for the first time that p11 in the serotonergic circuit as a key protein underlying the ability of ketamine to elicit a resilient stress-coping phenotype.

Chronic stress exposure is the most common finding underpinning the development of MDD, yet not all individuals exposed to stress develop this psychiatric condition [7, 54]. In this regard, understanding what makes individuals susceptible or resilient to stress, such as the molecular targets and brain circuitries, provide novel insights into how to prevent these psychiatric illnesses from developing [6]. Interestingly, rather than treating symptomatology in stress-related disorders, accumulating evidence has reported that ketamine is a pro-resilience agent by protecting against the development of stress-induced maladaptive behaviors in rodents [23, 25, 55] and humans [19–21, 56]. Here, our results revealed that a single injection of (*R,S*)-ketamine (15 mg/kg, i.p.) when administered 1 week before the onset of the restraint stress protocol, was effective in blunting the passive stress-coping behaviors induced by this stressor stimulus in the TST and SPT paradigms. Supporting our findings, previous studies demonstrated ketamine’s ability to protect against chronic restraint stress-induced depressive-like behavior in the TST, but also in the forced swim and splash tests, behavioral paradigms that assess behavioral despair and anhedonia, respectively [24, 57]. Additionally, our study provides novel evidence indicating that ketamine prophylactic administration elicits a resilient phenotype against chronic restraint stress-induced emotion discrimination impairment.

Although the molecular targets underlying the antidepressant effects of (R,S)-ketamine have been extensively investigated, the role of distinct brain regions and potential mechanisms underlying its protective actions remained to be fully uncovered [28]. Notably, prophylactic ketamine administration was found to elicit persistent changes in serotonergic DRN of mice challenged with uncontrollable stressor [29], however, the potential mechanisms within DRN that could contribute to its resilience-enhancing effects remains to be fully revealed. Therefore, to obtain deeper insights into the effects of ketamine in DRN under stress conditions, MALDI-MSI was conducted to spatially characterize neurotransmitters and their metabolites in this brain region [58, 59]. The results showed that stress protocol caused a significant reduction of the tryptophan metabolites 5HT, 5HTOL, and 6OHM, but also the dopamine pathway (DOPAL, HVA, and MOPEG) in DRN. Moreover, ketamine prophylactic administration, when given 1 week before the onset of stress protocol, buffered stress-induced reduction of 5HT and 5HTOL levels in DRN. This evidence presented here thus supports the conceptual idea that the ability of ketamine to prevent stress-induced alterations in the DRN serotonergic pathway including 5HT and its metabolite 5HTOL might be associated with the resilient stress-coping phenotype. Accordingly, it is noteworthy to mention that the serotonergic system is implicated in the antidepressant responses elicited by ketamine [60–62].

The multifunctional protein p11 is fundamental in the amplification of serotonergic signaling [30]. Indeed, p11 has been identified to be crucial to the physiopathology of MDD and responses to antidepressant strategies [30, 63]. Of note, our research group recently demonstrated that p11 is a potential predictor of (*R,S*)-ketamine response in patients with treatment-resistant MDD [43]. However, it is still unknown whether p11 modulation within DRN contributes to ketamine’s ability to promote a resilient phenotype against stress. Our data unveiled that chronic restraint stress procedure downregulated p11 mRNA levels in DRN, where this protein is highly enriched [36, 37]. This response agrees with previous findings reporting the ability of this model to elicit region-specific regulation on p11 levels [34, 35, 64]. Conversely, ketamine was effective in alleviating p11 reduction in the DRN induced by chronic restraint stress protocol when administered 1 week prior to the stressor stimulus. In support of these results, the ability of ketamine to be pro-resilient and protect against stressor-induced coping deficits and maladaptive behaviors was completely occluded in Sert-p11cKO mice. Additionally, behavioral studies of ketamine’s action have provided evidence for the recruitment of p11 in the long-lasting, but not rapid, antidepressant-like effects in chronically stressed mice [42]. Here, we now expand these findings by reporting that the modulation of p11 within DRN could play a role in the pro-resilient efficacy displayed by ketamine.

Compelling evidence has reported that p11 levels are reduced in brain tissue from patients diagnosed with MDD [31–33], whereas genetic p11 ablation induces depression-like behavioral traits [33, 36, 65]. These findings alongside the present results lead us to hypothesize that a reduction of p11 could be a critical event underlying not only resilience but also susceptibility to stress. Indeed, our experiments showed that although p11HET (representing a partial reduction of p11) displayed no basal behavioral alterations, they exhibited depression-like states in the EDT, TST, and SPT after the exposure to the subthreshold restraint stress, which is considered insufficient for producing a susceptible phenotype in naive mice [49]. These findings suggest that a global downregulation of p11 contributes to the susceptibility to stress-induced depression-like states. Further reinforcing this assumption, we also demonstrated that the global knockout of p11, as well as producing a basal depression-like phenotype as previously demonstrated [33, 34, 36], induces an amplified stress response and evokes a more pronounced depression-like state in the behavioral paradigms employed in this study. Indeed, a previous paper from our group demonstrated that p11 loss increases acute stress reactivity along with the HPA axis hyperresponsiveness [36]. Therefore, our findings support the notion that p11 deficiency could make mice more prone to stress-induced maladaptive behaviors.

Distinct brain circuits and cell-type specific regulation of p11 underlie the development of depressive-like phenotypes [33, 35, 36, 50–52, 64]. However, whether p11 in specific subtypes of neurons regulates susceptibility to stress remains to be fully investigated. Within this scenario, we next sought to identify which neuronal subtypes are involved in p11 deficiency-induced stress-susceptibility. Using a conditional knockout strategy, we deleted p11 specifically in Sert- or ChAT-expressing neurons, two circuitries that have been reported to exert an important role in p11 downregulation-induced depression-like states [33, 36, 41, 52]. The present report showed that the conditional knockout of p11 in both Sert- and ChAT-expressing neurons caused an impairment in emotion discrimination. Moreover, we also demonstrated that ChAT-p11cKO elicited a basal depressive-like phenotype, and this finding agrees with a prior report [52]. More importantly, our results uncovered that the specific knockout of p11 in Sert-, but not in ChAT-, expressing neurons induced passive stress-coping behaviors in the TST and SPT paradigms following a subthreshold restraint stress challenge. Therefore, one may suppose that while the loss of p11 in cholinergic neurons regulates basal depression-like phenotype, recapitulating global p11KO mice, p11 deficiency in serotonin neurons might contribute to the stress-susceptible outcomes in a task-dependent manner. Additionally, it is important to mention that because Sert-expressing neurons are also found in the gastrointestinal tract [66, 67] and these cells express p11 [68, 69], thus we cannot rule out the effects of gut and related peripheral pathways in the behavioral and neurochemical effects observed in Sert-p11cKO.

Because Sert-p11cKO mice showed stress susceptibility and Sert-expressing neurons in the brain mainly project from DRN [70], we examined neurotransmitters and their metabolites using MALDI-MSI in DRN of serotonergic-deficient p11cKO. MALDI-MSI analysis unveils that Sert-p11cKO mice displayed a significant reduction of metabolites related to tryptophan (5HT, 5HTOL, and 6OHM) and dopamine (HVA, NE, DOPEG, and MOPEG) pathway in DRN. These Sert-p11cKO-induced alterations in tryptophan/dopamine metabolism resemble those observed in chronically stressed mice, suggesting that p11 deficiency in serotonergic circuits might result in allostatic load and stress susceptibility. Thus, one may speculate that these serotonergic p11 deficiency-induced disturbances on tryptophan and dopamine metabolism in DRN could contribute to stress susceptibility. Accordingly, virus-mediated downregulation of p11 within the DRN induced a susceptible phenotype in p11^*flx/flx*^ mice, as evidenced by the passive stress-coping response in the TST and SPT after the subthreshold stress procedure. Furthermore, a recent study showed that p11 overexpression in DRN alleviated depression-like behavior caused by chronic stress [41]. These findings further reinforce the assumption that p11 within the DRN serotonergic circuit is responsible for regulating stress-relevant behaviors. Other than DRN, the MRN also projects to the brain and modulates the serotonergic circuitry. Sert-p11cKO mice present a trend of reducing p11 levels in MRN, which suggests that the remaining p11 mRNA expression is in non-Sert expressing cells. To differentiate the roles of p11 in DRN vs MRN in mediating the behavioral responses, we extended the analysis of p11^*flx/flx*^ mice injected with AAV-Cre aiming at DRN. We observed indeed a significant reduction of p11 levels in DRN, but not in the MRN. The virus-mediated downregulation of p11 in DRN resulted in stress-related behaviors which recapitulated the susceptible phenotype observed in Sert-p11cKO mice. These findings further reinforce the hypothesis that p11 within the DRN serotonergic circuit is responsible, at least in part, for regulating stress-relevant behaviors. However, we cannot rule out a participation of MRN in regulating a stress-related phenotype, but separate experiment targeting p11 in MRN would be necessary to address this issue.

Collectively, our data suggest that p11 deficiency regulates susceptibility to stress-induced depression-related phenotypes, and these behavioral maladaptations are dependent, at least in part, on p11 function in serotonergic neurons. Nevertheless, a major limitation of the current paper lies in the fact that we did not evaluate the activity of serotonergic neurons in the DRN of Sert-p11cKO mice under baseline and stress conditions, and this could have significance for interpreting the findings of the present study. Therefore, it is important to bear in mind that future studies investigating this topic are necessary to better understand how the p11 reduction within this circuit impacts stress-relevant behaviors. Our results also expand previous findings indicating that ketamine prophylactic administration buffers passive stress-coping behaviors induced by chronic stress exposure and demonstrated that this behavioral response was associated with p11 expression within DRN. The findings of the current study provide evidence for previously unknown mechanisms underlying the pro-resilience effects of ketamine, particularly by showing the modulation of p11 in serotonergic neurons as a key mediator of resilience-enhancing actions of this drug. Although the exact mechanism by which p11 regulates ketamine-induced pro-resilience remains to be characterized, one possibility is that ketamine may induce p11-dependent molecular alterations, including the modulation of p11 effector proteins 5-HT_1B_, 5-HT_4_, and mGluR_5_ receptors and/or ion channels [63]. Moreover, several studies have demonstrated that resilience-enhancing effects of (R,S)-ketamine and its related enantiomer (R)-ketamine, as well as metabolites (2R,6R)-HNK and (2S,6S)–HNK against a variety of stressors, might involve different targets [28], such as microRNAs, rapamycin protein complex 1 (mTORC1) signaling, and NOD-like receptor pyrin domain-containing 3 (NLRP3) inflammasome pathway [71–73]. Since the serotonin system is involved in a myriad of functions [74], the potential mechanisms or pathways already described in ketamine’s responses in addition to the current findings of this paper do not occlude one another, since parallel or complementary effects might regulate pro-resilience effects elicited by ketamine. Of note, another caveat of this study is that we only used female mice, thus future experiments are welcome to study sex-specific effects elicited by ketamine in serotonergic p11-deficient mice. Finally, the findings presented here may be translationally relevant, as recent studies show that ketamine might be a useful prophylactic agent against postpartum depression and depression comorbid with post-traumatic stress disorder [19–21, 56].

## Supplementary information

Supplemental material

## Data Availability

The data that support the findings of this study are available from the corresponding author upon reasonable request.
