# Supplementary material for: Enduring modulation of dorsal raphe nuclei regulates (R,S)-ketamine-mediated resilient stress-coping behavior
Source: Mol Psychiatry. 2024 Nov 26;30(6):2504–16. doi: 10.1038/s41380-024-02853-6 (PMC12092261; doi:10.1038/s41380-024-02853-6)
Supplement: Supplementary file 1 — Supplemental material [file 41380_2024_2853_MOESM1_ESM.docx]

Supplementary Information

**Enduring modulation of dorsal raphe nuclei regulates (R,S)-ketamine-mediated resilient stress-coping behavior**

Anderson Camargo^1^, Anna Nilsson^2^, Reza Shariatgorji^2^, Ellen Appleton^1^, Niclas Branzell^1^, Daniel Doyon^1^, Mattia Giovenzana^3^, Xiaoqun Zhang^1^, Daniel Dautan^1^, Per E. Andren^2^, Per Svenningsson^1^ *

1. Department of Clinical Neuroscience, Karolinska Institutet, Stockholm, Sweden.

2. Department of Pharmaceutical Biosciences, Uppsala University, Uppsala, Sweden.

3. Department of Medicine and Surgery, University of Milano Bicocca Monza, Italy.

* Corresponding authors

Dr. Anderson Camargo

Department of Clinical Neuroscience, Karolinska Institutet, Stockholm, Sweden.

E-mail address: anderson.camargo@ki.se

Dr. Per Svenningsson

Department of Clinical Neuroscience, Karolinska Institutet, Stockholm, Sweden.

E-mail address: per.svenningsson@ki.se

**Supplementary** **Material and Methods**

*Animals*

Rodents used in the current study included adult (8–12-week-old) female mice, because MDD prevalence is higher in women than in men [1] and females are more stress-sensitive than males [2, 3]. Wild-type (WT), constitutive global p11 knockout (p11KO), or p11 heterozygous (p11HET) mice were generated as previously described [4] on a C57BL/6J background. p11 floxed WT (p11*^flx/flx^*), conditional knockout of p11 in serotonin transporter (SERT)-expressing neurons (Sert-p11cKO, p11*^flx/flx^* Sert–Cre^+/-^), or conditional knockout of p11 in choline acetyltransferase (ChAT)-expressing neurons (ChAT-p11cKO, p11*^flx/flx^* ChAT–Cre^+/-^) mice were generated as previously described [4] on a C57BL/6J background. Mice were bred at the animal facility of Karolinska Institutet. Genotypes were confirmed by PCR. Mice were housed in groups of 4-5 in Type III Macrolon cages, under controlled temperature (21 ± 1 °C) and humidity (50 ± 20%) with a 12:12 h light/dark cycle (lights on at 7:00 a.m.), and with free access to food and water. All animals/samples were randomized. All experiments were approved by the Karolinska Institutet Ethical Committee (3218-2022) according to Swedish guidelines in full compliance with European requirements.

*Drugs*

(*R,S*)-ketamine was purchased from Sigma-Aldrich (St. Louis, MO), dissolved in sterile saline (0.9% NaCl), and administered via intraperitoneal (i.p.) route at a dose of 15 mg/kg. Ketamine was freshly prepared before administration and administered in a volume of 10 ml/kg body weight. Mice received a single i.p. administration of ketamine (15 mg/kg) and were subjected to a 1-week washout period before the exposure to stress protocol [5, 6].

*Repeated restraint stress protocol*

To perform the chronic restraint stress protocol, mice were subjected to immobilization (2 h/day, for 14 days) using a 50 ml falcon tube. The stress protocol restrained all physical movement without submitting the animal to pain [7]. On the testing day, 24 h after the last restraint stress episode, mice were subjected to behavioral tests. In another experimental approach, mice were exposed to restraint stress (2 h/day) for 7 days, which has been reported to be a submaximal restraint stress procedure insufficient for producing a susceptible phenotype in naïve mice [8]. Mice underwent immobilization using a 50 ml falcon tube and 24 h after the last stress episode, mice were subjected to behavioral tests.

*Stereotaxic surgical procedures for viral infusions*

All surgeries were performed under aseptic conditions. Mice were deeply anesthetized with isoflurane (1.5 to 4% in O2) and placed in a stereotaxic apparatus (Kopf Instruments). Ophthalmic ointment was applied. Following shaving and skin incision, a small cranial hole was made above the targeted structure. All coordinates were obtained relative to the bregma and dorsoventral coordinates were from the brain surface. Viral injections were performed using a nanos-syringe (#7001, Hamilton Syringes) at 50 nl/min using a micro syringe pump (micro4, WPI). After completion of the injection, the syringe was maintained in position for 10 min prior to withdrawing to reduce backfilling. At the end of the surgery, animals were given injections of Buprenorphine (0.1mg/kg, i.p.) and Baytril (0.05mg/kg, i.p). Viruses used for all experiments are as follows: AAV5-CaMKIIa-GFP (control virus) and AAV5-CaMKIIa-GFP-Cre (viral expression of Cre under CaMKIIa promoter, UNC Vector Core, #6450B, titer 10^−12^). Viruses (titer 3.5 x 10^-12^) were injected in the DRN (coordinates AP: −4.6, ML: ±0.05, DV: −3.3). A total volume of 100 nl was infused per animal. At the end of behavioral experiments, all injections were confirmed post hoc using immunostaining for GFP, and animals with misplaced injections were discarded.

*Behavioral tests*

All tests were conducted at least 4 weeks after virus infusion. Animals were habituated to experimental conditions prior to behavioral tests and underwent behavioral testing as follows: emotion discrimination test, sucrose preference test, open-field test, and tail suspension test, 24 h apart. Mice were randomly assigned to the treatment groups and observers were blinded to treatments and genotypes during the experiments and behavioral analysis. After the behavioral tests (24 h), mice were euthanized by decapitation and the brains were collected and snap-frozen in isopentane, cooled in dry ice, and subsequently stored at -80°C.

*Emotion discrimination test (EDT)*

The emotional discrimination test was performed as described previously [9, 10]. Briefly, testing mice (observers) were habituated (6 min) inside a custom-made 3 chambers box equipped with a dark separator between two cylindrical cups that hosted the demonstrators [10]. The separator (11 × 14 cm) between the two cups was wide enough to cover the reciprocal view of the demonstrators while leaving the observer mice free to move between the two sides of the cage. On the day of the experiments, the observer was placed in the box for 6 minutes for acclimatization, then 2 stimulus mice were placed in a 5 cm radius plexiglass cup with holes drilled to allow contact. Both cups were placed on either side of the 3-chamber apparatus with no direct reciprocal view between demonstrators. Immediately following the placement of the 2 stimulus mice, the trial was recorded for 6 minutes. The first test was done with two naïve WT mice (Neutral *versus* Neutral), matched by sex and age to the observer) and used to determine any possible bias (habituation). Next, both stimulus and cups were replaced with 2 fresh stimuli that either presented a neutral state (WT mice) or a stressed state (WT mice that underwent restraint stress using an individual rodent restraint device made of Plexiglas fenestrate for 15 min before the beginning of the trial). These mice were then immediately moved to the testing arena and then the trial was recorded for 6 minutes [9]. The cups were replaced after each subject with clean copies to avoid scent carryover. The side for each stimulus was randomly assigned across experiments. The chambers were wiped with 75% ethanol and allowed to air dry between tests. During both habituation and behavioral testing, the apparatus was placed in a dim light condition (~10 lux). Habituation and test videos were collected using a high-resolution camera located above the apparatus, connected to Ethovision XT tracking software (Noldus). The videos were then scored offline by an experimenter blind to the manipulations, in which the time sniffing (defined by the time the observer initiated sniffing toward the stimulus) was extracted using custom keyboard software. The time spent sniffing was expressed selectively in the first 2 min of the task, which is in line with previous studies demonstrating that the ability to discriminate was detected in the first 2 minutes and then gradually waned [9, 10]. Discrimination index calculation was based on the following formula: time spent sniffing time the stressed mouse = (time spent sniffing time the neutral mouse/time spent sniffing time the stressed mouse).

*Sucrose preference test (SPT)*

The sucrose preference index was measured as previously proposed [11]. Mice were individually housed and had ad libitum access to two bottles (one containing water and the other containing a 1,5 % sucrose solution) for a period of 24 h. The consumption of sucrose was calculated as a percentage of sucrose solution consumed relative to the total amount of liquid drunk. The sucrose preference was used as a measurement of stress-induced anhedonia-like behavior (hyposensitivity to pleasure marker) in mice.

*Open-field test (OFT)*

The locomotor activity was measured in the open field of a Plexiglas chamber (46 × 46 × 46 cm) with a grey floor and walls. The arena was illuminated by reflected light, providing an intensity of 30 Lux on the floor of the arena. Each mouse was placed in the corner of the open field, and locomotion was recorded for the indicated period of 6 min [4]. The arena was cleaned with 70% ethanol after each test session to eliminate olfactory cues. Video tracking was performed using a video camera mounted in the ceiling and analyzed by EthoVision XT11.5 (Noldus) software.

*Tail suspension test (TST)*

The total immobility time of mice suspended by the tail was measured as previously proposed [12]. Visually isolated mice were suspended 50 cm above the floor by adhesive tape placed approximately 1 cm from the tip of the tail. Immobility time was recorded for 6 min by an experienced observer blind to the experimental groups. Mice were considered immobile only when they hung passively and completely motionless. The immobility time in the tail suspension test was taken as indicative of antidepressant-like or depressogenic-like effects.

*Radioactive in situ hybridization*

Cryostat (Leica CM 3050 S) fresh frozen sections (12 μm thick) from the cortex, striatum, and dorsal raphe nuclei were fixed in 4% paraformaldehyde (Sigma-Aldrich, St. Louis, MO, USA) and hybridized with 35S-radiolabeled antisense riboprobes against p11 as previously described [4]. After hybridization, slides were exposed to Kodak Biomax maximum resolution film (Carestream, Rochester, NY), in room temperature for 21 days prior to development. Densitometric measurements of mRNA expression were obtained from digitized autoradiograms using FIJI software and are presented as optical density values compared to the control group 100%.

*Fluorescent in situ hybridization (RNA Scope)*

Fluorescent *in situ* hybridization was performed using the RNAscope Multiplex Fluorescent Assay (Advanced Cell Diagnostics, Abingdon, Oxford). Cryostat (Leica CM 3050 S) fresh frozen sections (12 μm thick) were post fixed in 4% PFA for 15 min at 4°C and dehydrated in graded alcohols. Next, Protease IV (Advanced Cell Diagnostics) was applied for 30 min at room temperature. Sections were then hybridized with the probes: p11 (MmS100a10, # 410901) for 2 h at 40 °C [4]. The hybridization step was followed by standardized steps of amplification (Amp 1-FL 30 min at 40 °C, Amp 2-FL 15 min at 40 °C, Amp 3-FL 30 min at 40 °C, Amp 4C-FL 15 min at 40 °C). After the last amplification step, sections were counterstained with DAPI (Advanced Cell Diagnostics), and immunostained with a Tph primary antibody (1:500, #AB1541, Merck, Solna, Sweden) or ChAT primary antibody (1:500, #AB144P, Merck, Solna, Sweden). After 24 h incubation, sections were washed, and incubated with Alexa Fluor 568- or 647-conjugated secondary antibodies (1:500, #A21448, #A11057 Invitrogen, Stockholm, Sweden), then mounted with Dako fluorescent mounting medium (Agilent Technologies, Kista, Sweden). Sections were imaged on a Carl Zeiss LSM 880 confocal microscope (Carl Zeiss AB, Stockholm, Sweden) using 20x objective. Z-stacks of 7-10 µm thickness were obtained in each caption [4, 13]. Quantification of expression of p11 was determined as the fluorescence intensity of signal dots within the entire DRN or MRN region or within individual cells using FIJI software. The integrated fluorescent intensity is expressed as compared to the control group 100%. Moreover, using macro automated counter on FIJI software, we also determined the fluorescent intensity per ROI at concentric 25 pixels x 2.4 μm intervals throughout the sections, and the fluorescent intensity was normalized using z-score function.

*Immunofluorescence and viral infection efficiency*

Coronal sections (12 µm thick) were collected using a cryostat sectioner, and thaw-mounted on Polysine® glass slides (VWR, Stockholm, Sweden). Sections were fixed in 4% paraformaldehyde (Sigma-Aldrich, St. Louis, MO, USA) and washed three times with 0.1 M PBS containing 0.1% Triton X-100 (PBS-T) for 15 min each time, and permeabilized with PBST in 2% normal goat serum, 2% normal horse serum for 1 h. After blocking, sections were incubated in the primary solution containing PBS-Triton, 1% NGS and the corresponding primary antibody overnight at 4 ºC under constant shaking. The immunohistochemistry was done using the antibody against GFP (to enhance YFP staining, made in Rabbit, already conjugated to Alexa-488, concentration 1:1000, Thermofisher, #A21311). Slices were washed three more times in PBS-T for 15 min and coverslipped with Vectashield mounting medium with DAPI (Vector Laboratories, Newark, CA, USA). All the sections were examined under a Zeiss LSM710 confocal microscope (Carl Zeiss AB, Stockholm, Sweden). All histology findings were confirmed in at least six different animals.

*Matrix-assisted laser desorption/ionization-mass spectrometry imaging (MALDI-MSI)*

MALDI-MSI analysis of neurotransmitters in DRN was performed as previously reported [14]. Fresh frozen mouse brains were cut at a thickness of 12 μm using a cryostat sectioner, thaw-mounted onto conductive indium tin oxide (ITO) glass slides (Bruker Daltonics) and stored at −80 °C. To minimize enzymatic degradation, sections were thawed and dried in a vacuum desiccator for 20 min. Reactive matrix, FMP-10, was dissolved in 5.5 ml of 70% acetonitrile (4.4 mM) and sprayed over the tissues using an automated pneumatic sprayer (TM-Sprayer, HTX Technologies). The nozzle temperature of the sprayer was set at 90 °C, and the reagent was sprayed pneumatically (6 psi of N2) onto the sample in twenty horizontal passes at a linear nozzle velocity of 110 cm/min with 2 mm track spacing and a flow rate of 80 μl/min. Prior to MSI analysis, the slide was scanned on a flatbed scanner (Epson perfection V500). All MALDI-MSI and experiments were performed in positive ionization mode using a MALDI-FTICR (solariX 7 T 2ω, Bruker Daltonics) mass spectrometer equipped with a Smartbeam II 2 kHz laser. Data were acquired within the range of m/z 150–1000 by firing 100 laser shots per raster position and Q1 mass was set to m/z 379. The lateral resolution was 100 µm. The method was externally calibrated using red phosphorus and internally calibrated using the FMP-10 cluster ion signal (m/z 555.2231) as lock mass. The identification of neurotransmitters and metabolites has been described elsewhere [15, 16]. It is important to mention that we attempted to further verify the identification of 5HTOL via MS/MS analysis, but unfortunately, this was unsuccessful due to the low abundance. All chemicals and solvents were purchased from Sigma-Aldrich and were used without further purification. Reactive matrix for the detection of neurotransmitters (FMP-10) was purchased from Tag-ON AB (Uppsala, Sweden). DRN was annotated according to Paxinos and Franklin’s stereotaxic atlas. Data were normalized against the root mean square (RMS) of all data points and visualized using flexImaging (v. 5.0, Bruker Daltonics). Numerical data (m/z and intensity values) for DRN were exported using SCiLS Lab (v. 2019a Pro, Bruker Daltonics). The maximum ion intensity values of the average spectra from the DRN of each individual for all identified neurotransmitters and metabolites were exported from SCiLS for statistical analysis. The average intensity values per brain area were log-transformed.

*Statistical analysis*

All statistical analyses were done using GraphPad Prism (GraphPad, San Diego, CA, USA). Outlier detection within each data set was assessed using Grubb’s test calculator tool from GraphPad and identified outliers were removed. The D’Agostino-Pearson test was used to assess data normality. The differences among experimental groups were determined by two-tailed unpaired Student’s t-test or two-way analysis of variance (ANOVA) followed by Tukey’s post hoc test, when appropriate. Differential abundance analysis of neurotransmitters and metabolites (shown in volcano plots) were performed using independent t-tests and the Benjamini-Hochberg multiple-testing correction to control the false discovery rate (FDR<0.05). All experiments were performed in a randomized manner, the experimenters were blinded, and the number of animals was chosen based on previous studies from our group. We did not assume sphericity and we used Geisser-Greenhouse correction, as recommended by Prism. The details of statistical tests and their outcomes are presented in the Supplementary Information. Data are presented as mean ± standard error of the mean (SEM). A value of P<0.05 was considered significant.

**Supplementary References**

1. World Health Organization. Depression and other common mental disorders: Global health estimates. *World Health Organization*. 2017:1–24.

2. Shepard R, Page CE, Coutellier L. Sensitivity of the prefrontal GABAergic system to chronic stress in male and female mice: Relevance for sex differences in stress-related disorders. *Neuroscience* 2016; 332:1–12.

3. Marrocco J, Petty GH, Ríos MB, Gray JD, Kogan JF, Waters EM, et al. A sexually dimorphic pre-stressed translational signature in CA3 pyramidal neurons of BDNF Val66Met mice. *Nat Commun* 2017; 8:808.

4. Sousa VC, Mantas I, Stroth N, Hager T, Pereira M, Jiang H, et al. P11 deficiency increases stress reactivity along with HPA axis and autonomic hyperresponsiveness. *Mol Psychiatry* 2020; 26:3253–3265.

5. Brachman RA, McGowan JC, Perusini JN, Lim SC, Pham TH, Faye C, et al. Ketamine as a prophylactic against stress-induced depressive-like behavior. *Biol Psychiatry* 2016;79:776–786.

6. Camargo A, Torrá ACNC, Dalmagro AP, Valverde AP, Kouba BR, Fraga DB, et al. Prophylactic efficacy of ketamine, but not the low-trapping NMDA receptor antagonist AZD6765, against stress-induced maladaptive behavior and 4E-BP1-related synaptic protein synthesis impairment. *Prog Neuropsychopharmacol Biol Psychiatry* 2022; 115:110509.

7. Seo JS, Wei J, Qin L, Kim Y, Yan Z, Greengard P. Cellular and molecular basis for stress-induced depression. *Mol Psychiatry* 2016:1–8.

8. Shui Y, Wang L, Luo X, Uchiumi O, Yamamoto R, Sugai T, et al. Homer1a disruption increases vulnerability to predictable subtle stress normally sub-threshold for behavioral changes. *Brain Res* 2015; 1605:70–75.

9. Ferretti V, Maltese F, Contarini G, Nigro M, Bonavia A, Huang H, et al. Oxytocin signaling in the central amygdala modulates emotion discrimination in mice. *Curr Biol* 2019; 29:1938-1953.

10. Scheggia D, Managò F, Maltese F, Bruni S, Nigro M, Dautan D, et al. Somatostatin interneurons in the prefrontal cortex control affective state discrimination in mice. *Nat Neurosci* 2020; 23:47–60.

11. Liu M-Y, Yin C-Y, Zhu L-J, Zhu X-H, Xu C, Luo C-X, et al. Sucrose preference test for measurement of stress-induced anhedonia in mice. *Nat Protoc* 2018; 13:1686–1698.

12. Steru L, Chermat R, Thierry B, Simon P. The tail suspension test: A new method for screening antidepressants in mice. *Psychopharmacology* 1985; 85:367–370.

13. Skiteva O, Yao N, Mantas I, Zhang X, Perlmann T, Svenningsson P, et al. Aberrant somatic calcium channel function in cNurr1 and LRRK2-G2019S mice. *NPJ Parkinsons Dis* 2023; 9:56.

14. He Y, Kaya I, Shariatgorji R, Lundkvist J, Wahlberg LU, Nilsson A, et al. Prosaposin maintains lipid homeostasis in dopamine neurons and counteracts experimental parkinsonism in rodents. *Nature Commun* 2023; 14:1–22.

15. Shariatgorji R, Nilsson A, Fridjonsdottir E, Strittmatter N, Dannhorn A, Svenningsson P, et al. Spatial visualization of comprehensive brain neurotransmitter systems and neuroactive substances by selective in situ chemical derivatization mass spectrometry imaging. *Nat Protocols* 2021; 16:3298–3321.

16. Shariatgorji M, Nilsson A, Fridjonsdottir E, Vallianatou T, Källback P, Katan L, et al. Comprehensive mapping of neurotransmitter networks by MALDI–MS imaging. *Nat Methods* 2019; 16:1021–1028.

**Supplementary figures and legends**


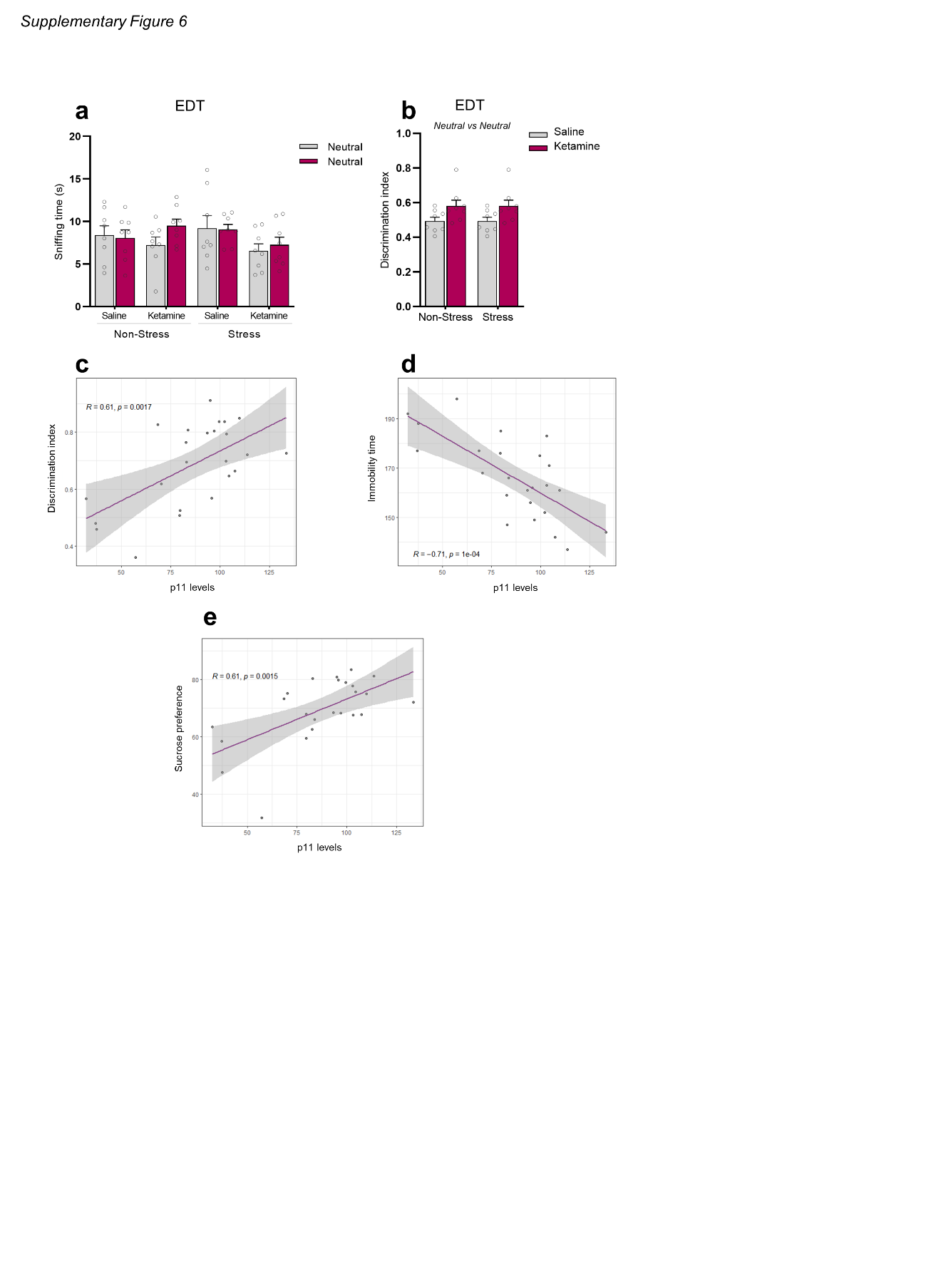


**Supplementary Figure 1.** Effects of prophylactic administration of ketamine in the emotion discrimination test (EDT). (**a**) No significant effects were detected in the time sniffing of saline- or ketamine-treated WT mice subjected to the EDT in a neutral *versus* neutral condition. (**b**) No significant effects were observed in the discrimination index of saline- or ketamine-treated WT mice, regardless of stress protocol. Values are expressed as means ± S.E.M (n = 8). Individual data are represented as dots (two-way ANOVA).


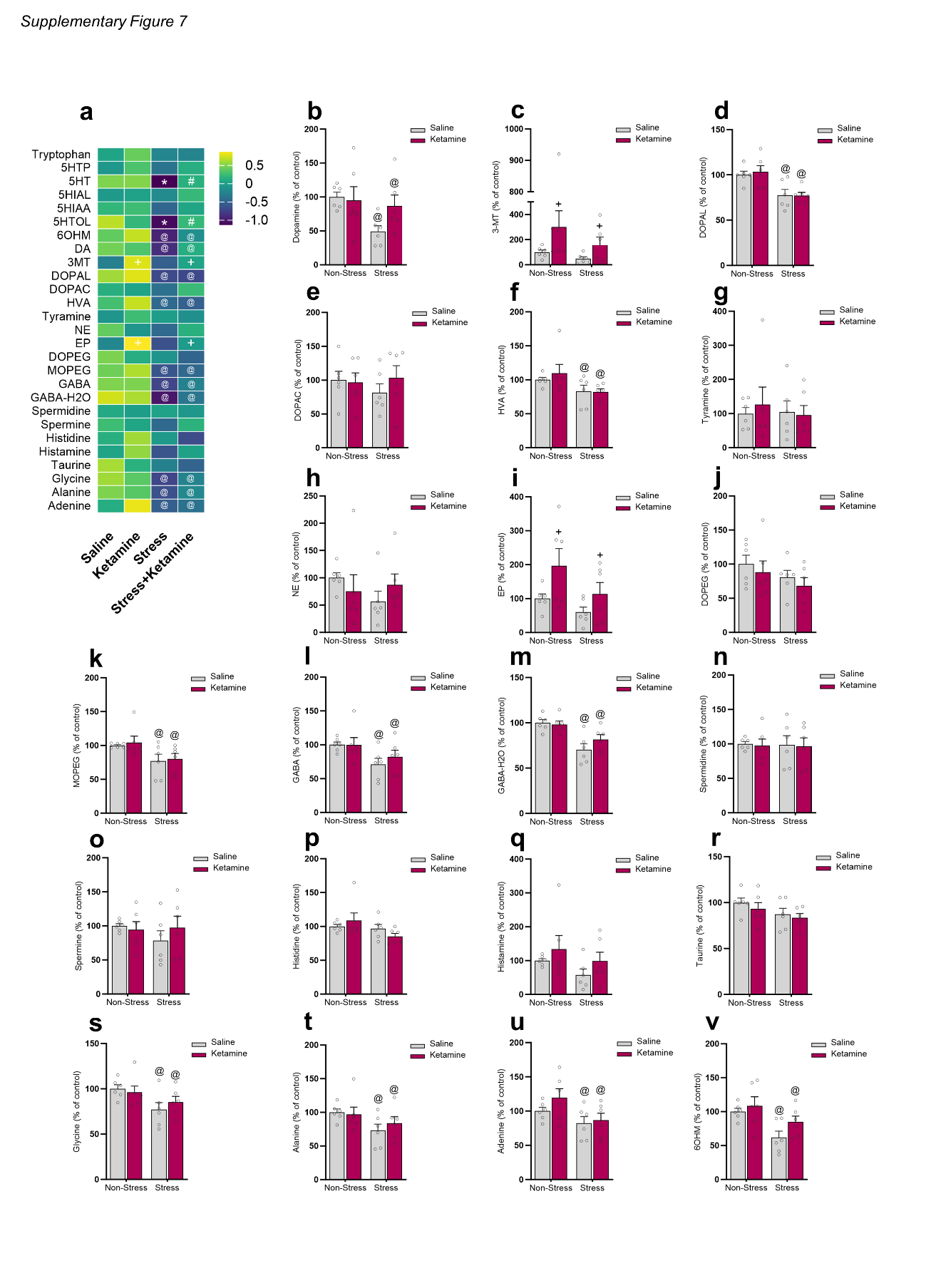


**Supplementary Figure 2.** Effects of ketamine prophylactic administration against stress-induced neurotransmitter and metabolites alterations in DRN. (**a**) Heat map of neurotransmitters and their metabolites in DRN of non-stressed or stressed mice treated with saline or ketamine. Each square represents mean of the corresponding analyte with the value normalized across the groups. Bar graph showing the quantification of the peaks of dopamine (**b**), 3MT (**c**), DOPAL (**d**), DOPAC (**e**), HVA (**f**), tyramine (**g**), NE (**h**), EP (**i**), DOPEG (**j**), MOPEG (**k**), GABA (**l**), GABA-H2O (**m**), spermidine (**n**), spermine (**o**), histidine (**p**), histamine (**q**), taurine (**r**), glycine (**s**), alanine (**t**), adenine (**u**), and 6OHM (**v**). Values are expressed as means ± S.E.M (n = 6). Individual data are represented as dots. * *p* < 0.05 compared with the non-stressed saline-treated group; # *p* < 0.05 compared with stressed saline-treated group (two-way ANOVA followed by Tukey’s post hoc test). @ *p* < 0.05 compared with non-stressed mice (i.e., a significant main effect of stress protocol); + *p* < 0.05 compared with saline-treated mice subjected or not to stress protocol (i.e., a significant main effect of ketamine treatment).


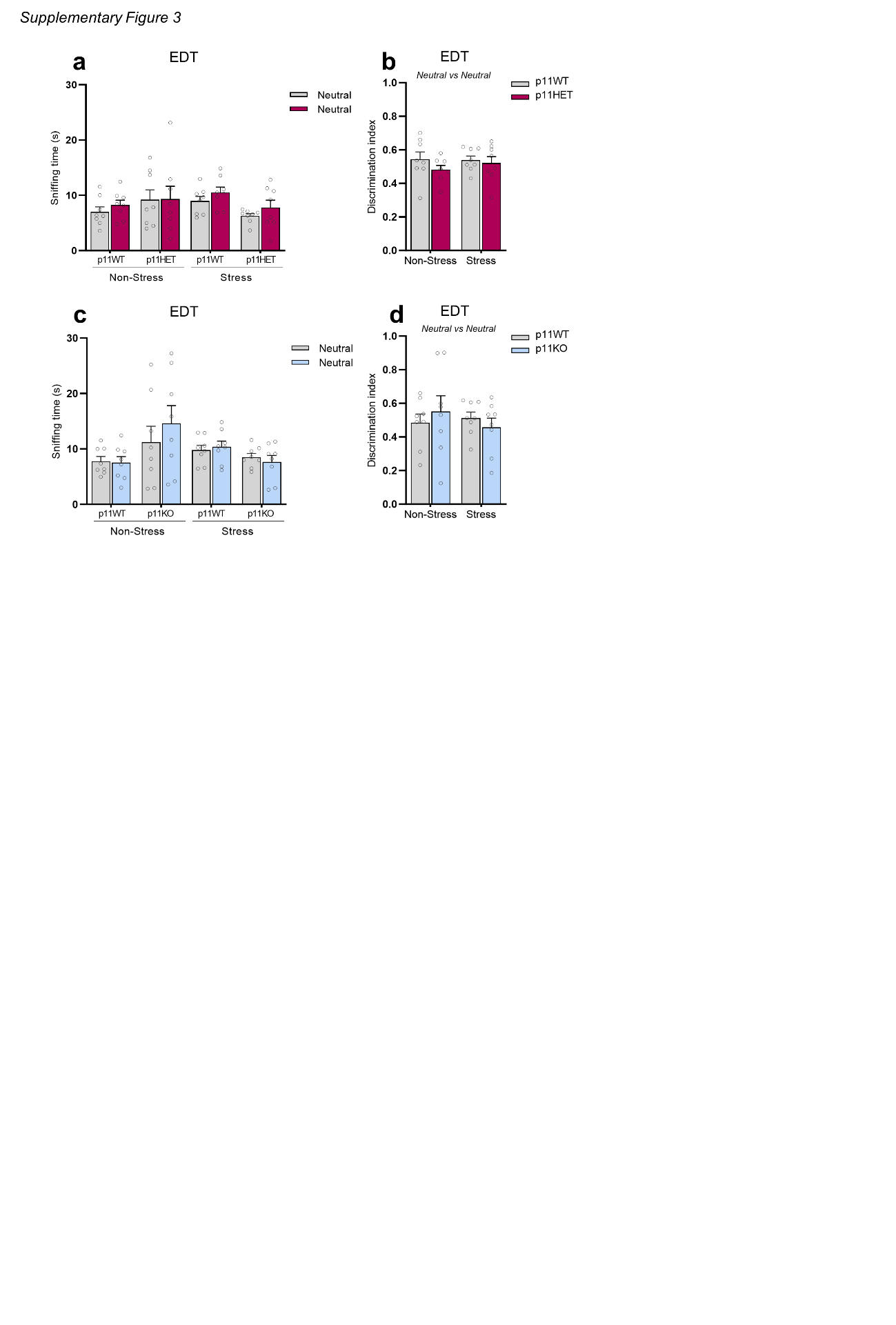


**Supplementary Figure 3.** Effects of p11 downregulation (p11HET) and global p11 knockout (p11KO) in the emotion discrimination test (EDT). (**a**) No significant bias was observed in the time sniffing of p11HET mice subjected to the EDT in a neutral *versus* neutral condition. (**b**) No significant effects were observed in the discrimination index of p11HET. (**c**) There were no significant differences observed in time sniffing p11KO mice subjected to the EDT in a neutral *versus* neutral condition. (**d**) All groups presented a comparable discrimination index. Values are expressed as means ± S.E.M (n = 8). Individual data are represented as dots. Data were analyzed by two-way ANOVA.


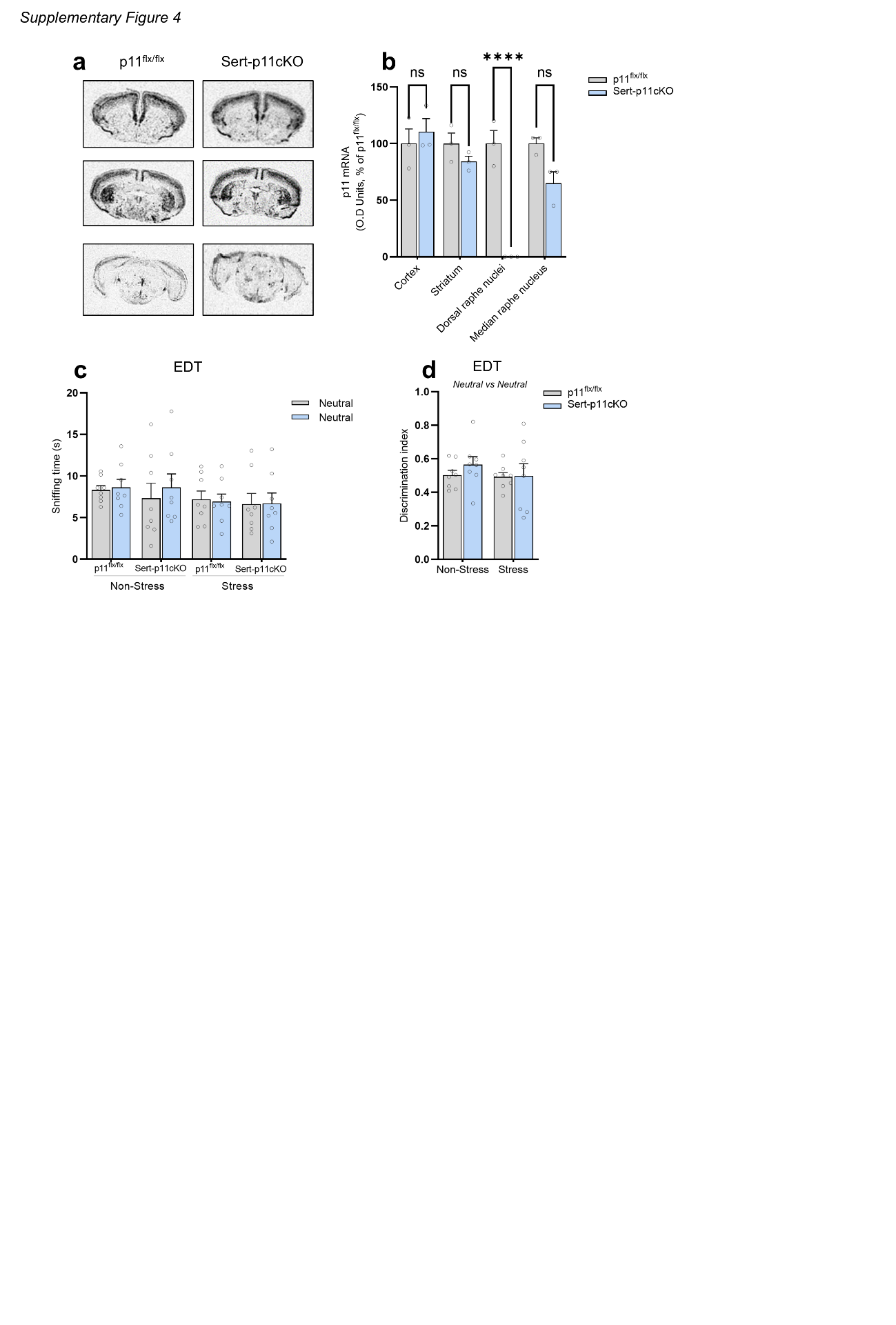


**Supplementary Figure 4.** Effects of conditional knockout of p11 in Sert-expressing neurons (Sert-p11cKO) in different brain regions and in the emotion discrimination test (EDT). (**a**) Representative *in situ* hybridization images illustrating p11 mRNA in the cortex, striatum, dorsal raphe nuclei, and median raphe nucleus of p11*^flx/flx^* and Sert-p11cKO. (**b**) Quantification of p11 mRNA in the cortex, striatum, dorsal raphe nuclei, and median raphe nucleus of p11*^flx/flx^* and Sert-p11cKO. (**c**) No significant bias was observed in the time sniffing of Sert-p11cKO mice subjected to the EDT in a neutral *versus* neutral condition. (**d**) No significant effects were observed in the discrimination index of Sert-p11cKO. Values are expressed as means ± S.E.M (n = 3-8). Individual data are represented as dots. *** *p* < 0.01 compared with the non- p11*^flx/flx^* group (two-way ANOVA followed by Tukey’s post hoc test).


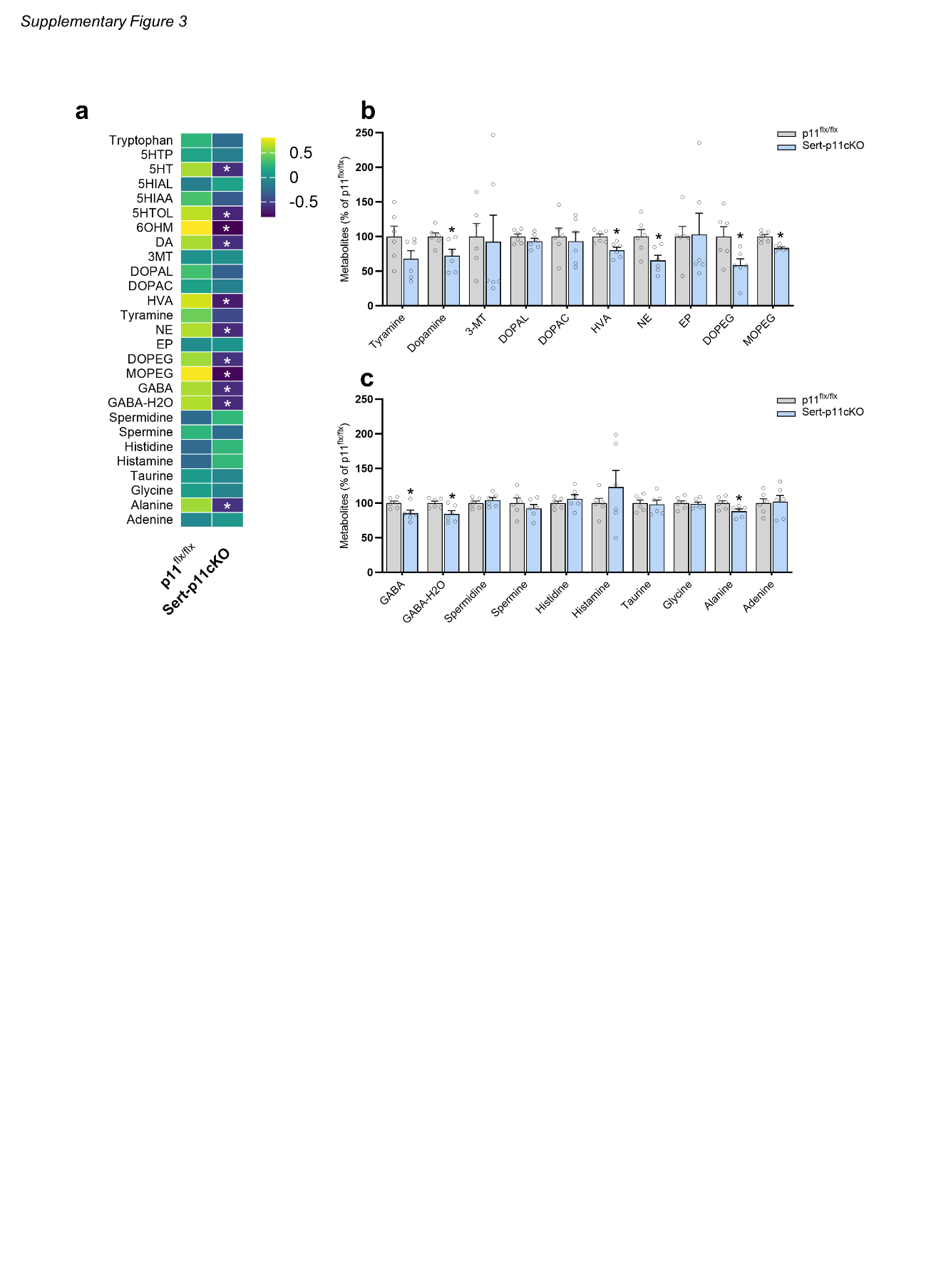


**Supplementary Figure 5.** Serotonergic p11-deficient mice present neurotransmitter and metabolites alterations in DRN. (**a**) Heat map of neurotransmitters and their metabolites in DRN of p11*^flx/flx^* and Sert-p11cKO mice. Each square represents mean of the corresponding analyte with the value normalized across the groups. (**b**) Bar graph showing the quantification of the dopamine and metabolite peaks. (**c**) Bar graph showing the quantification of the peaks of GABA, GABA-H2O, spermidine, spermine, histidine, histamine, taurine, glycine, alanine, and adenine. Values are expressed as means ± S.E.M (n = 6). Individual data are represented as dots. * *p* < 0.05 compared with the p11*^flx/flx^* group (Student's t-test).


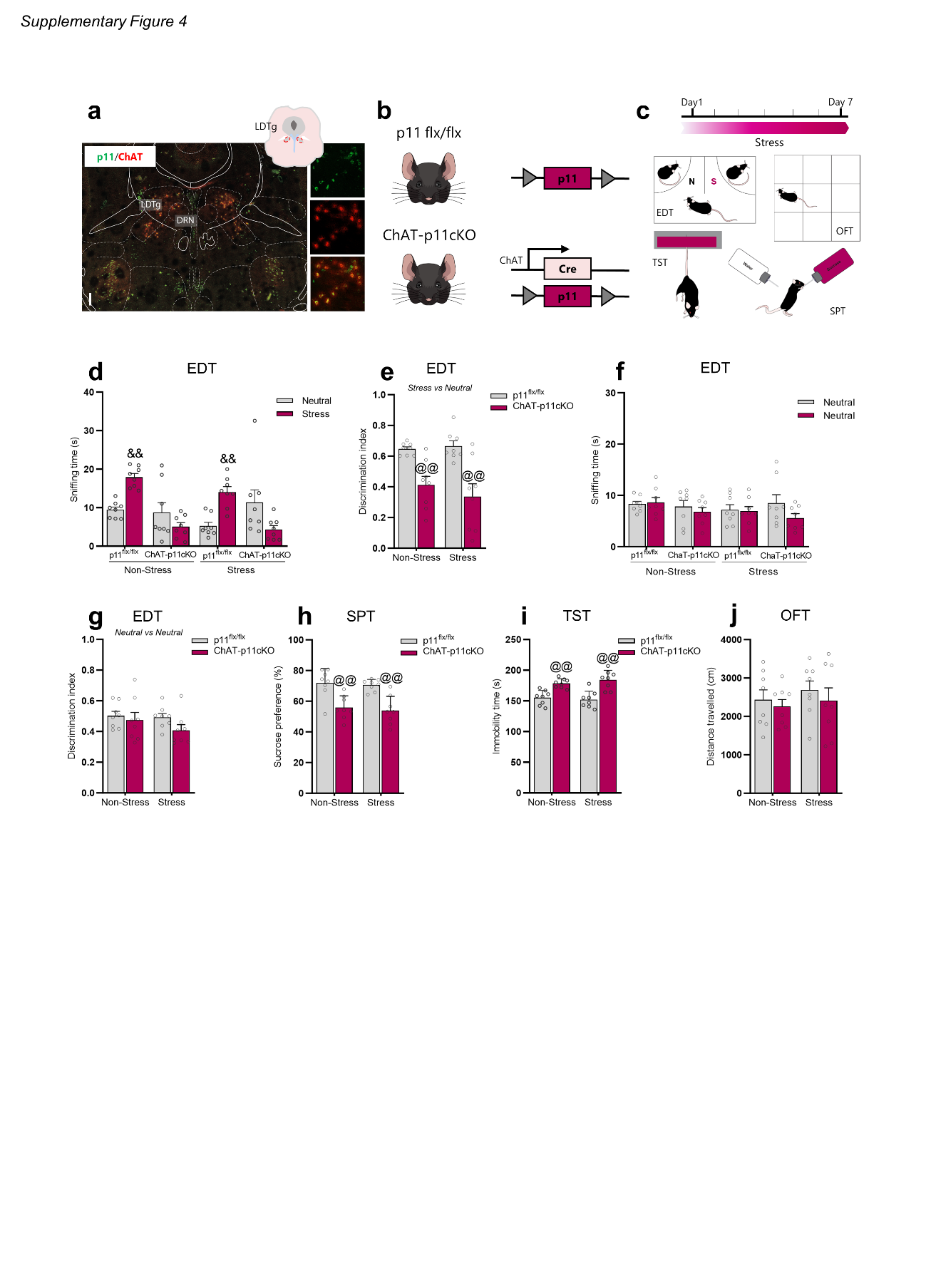


**Supplementary Figure 6.** Behavioral alterations of conditional knockout of p11 in ChAT-expressing neurons. (**a**) Representative RNA scope image illustrating p11 transcripts (green) and ChAT-expressing neurons (red) in the laterodorsal tegmental nucleus (LDTg). Scale bar: 100 µm. (**b**) Schematic depiction of transgenic ChAT-p11cKO (p11*^flx/flx^* ChAT–Cre^+/-^) mouse construct. (**c**) Experimental time plan and behavioral tests. (**d**) ChAT-p11cKO elicited reduced time sniffing the stressed conspecific when compared to the neutral one in the emotion discrimination test (EDT). (**e**) ChAT-p11cKO mice presented an impaired discrimination index. (**f**) No significant bias was observed in the time sniffing of ChAT-p11cKO mice subjected to the EDT in a neutral *versus* neutral condition. (**g**) No significant effects were observed in the discrimination index of ChAT-p11cKO in a neutral *versus* neutral condition. (**h**) ChAT-p11cKO elicited decreased sucrose preference in the sucrose preference test (SPT), regardless of stress protocol. (**i**) ChAT-p11cKO mice presented increased immobility in the tail suspension test (TST), regardless of stress protocol. (**j**) All groups had comparable distance traveled in the open-field test (OFT). Values are expressed as means ± S.E.M (n = 8). Individual data are represented as dots. && *p* < 0.01 compared with the neutral stimulus (two-way ANOVA followed by Tukey’s post hoc test). @@ *p* < 0.01 compared with p11*^flx/flx^* mice subjected or not to stress procedure (i.e., a significant main effect of genotype).


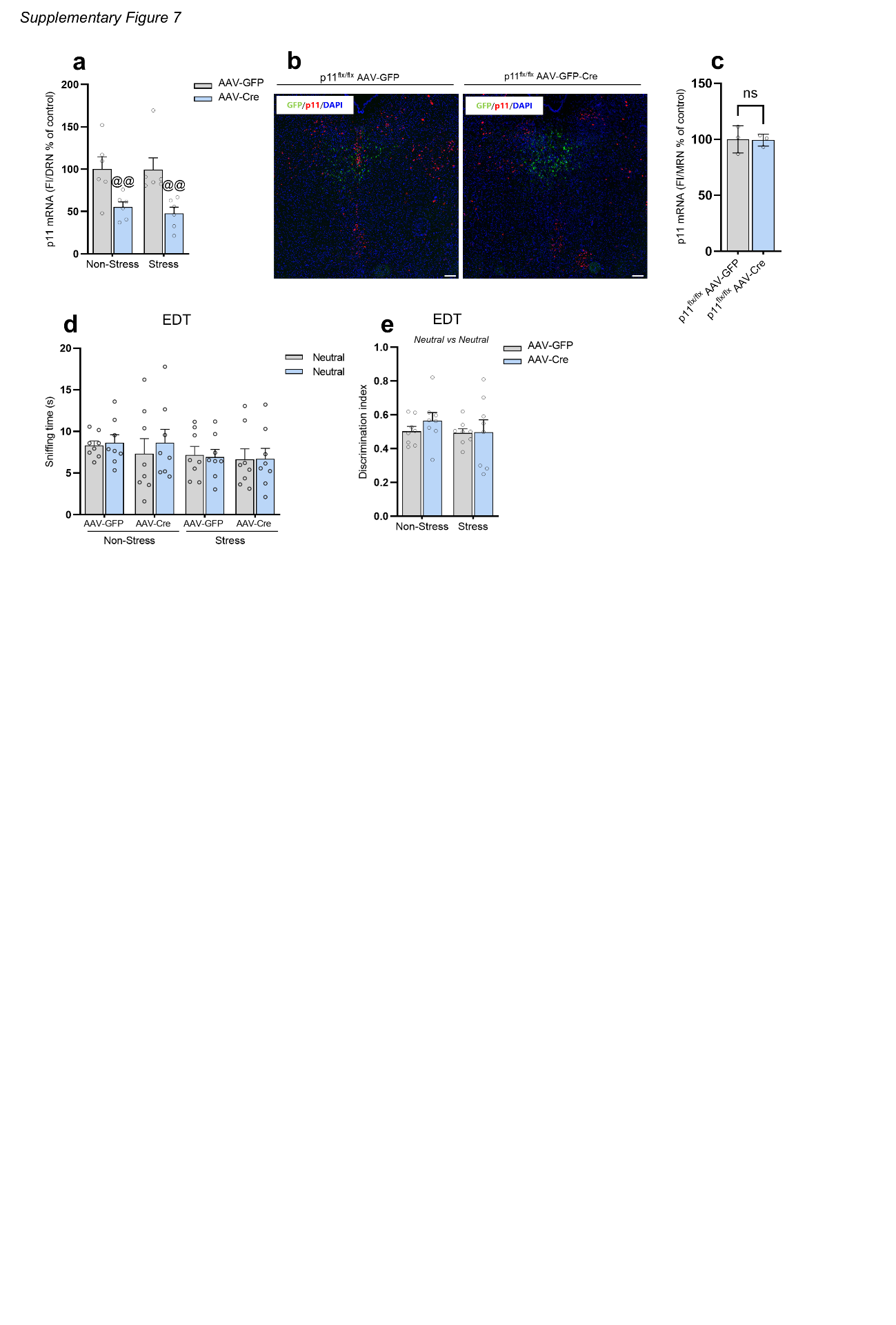


**Supplementary Figure 7.** Effects of viral-mediated p11 downregulation in the emotion discrimination test (EDT). (**a**) p11 levels in DRN of p11*^flx/flx^* mice injected with GFP or GFP-Cre subjected or not to stress procedure. (**b**) Representative image of AAV-GFP and AAV-GFP-Cre virus expression (green), p11 levels (red), and DAPI (blue) staining in the DRN and MRN from p11*^flx/flx^* mice. (**c**) p11 levels in MRN of p11*^flx/flx^* mice injected with GFP or GFP-Cre. (**d**) No significant effects were found in the time sniffing of AAV-GFP- or AAV-Cre-injected mice subjected to the EDT in a neutral *versus* neutral condition. (**e**) No significant effects were observed in the discrimination index of AAV-GFP- or AAV-Cre-injected mice. Values are expressed as means ± S.E.M (n = 3-8). Individual data are represented as dots. Data were analyzed by two-way ANOVA followed by Tukey’s post hoc test or Student’s t-test. @@ *p* < 0.01 compared with GFP-injected p11*^flx/flx^* mice subjected or not to stress procedure (i.e., a significant main effect of AAV-Cre injection).


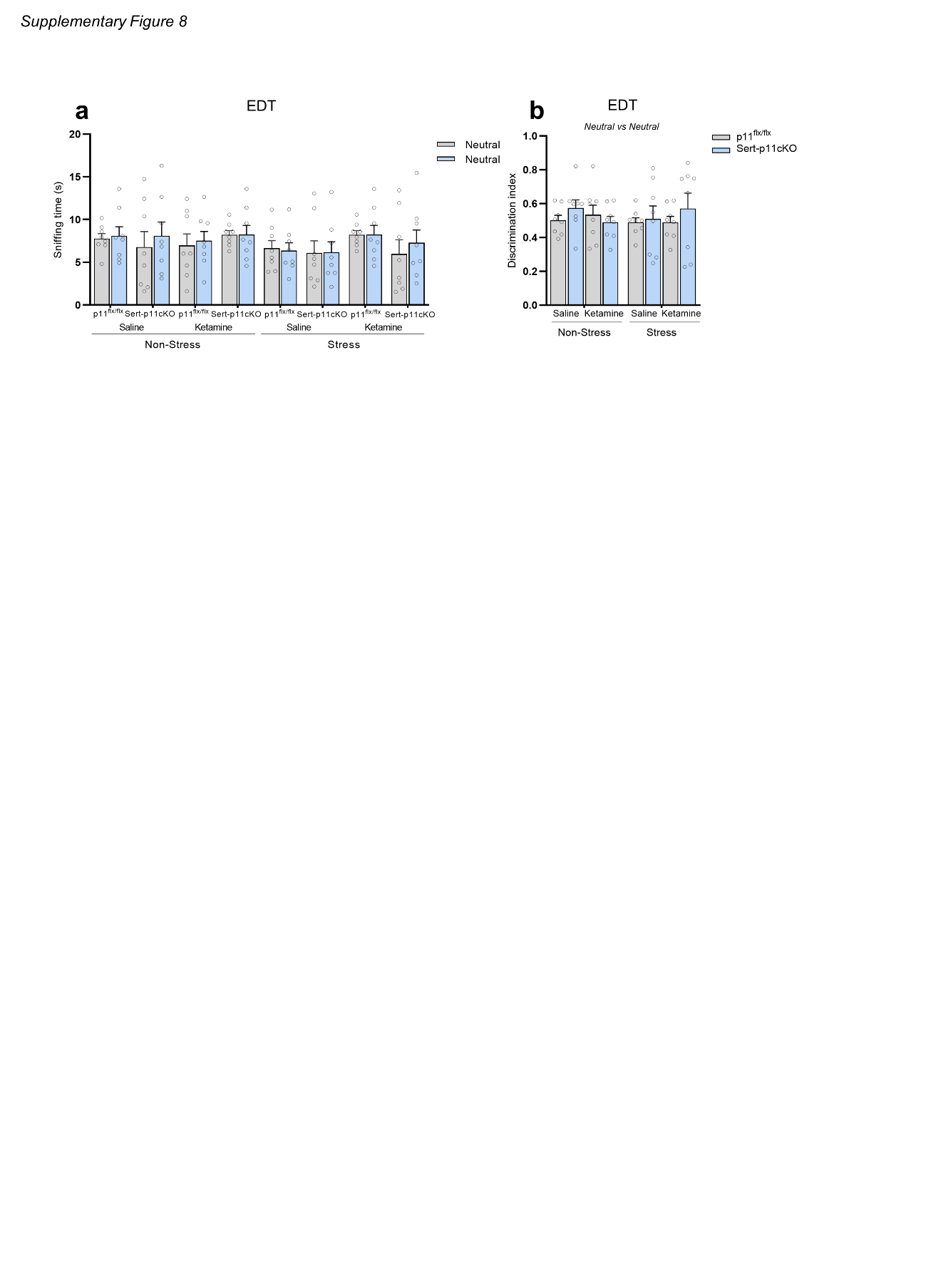


**Supplementary Figure 8.** Effects of prophylactic administration of ketamine in p11*^flx/flx^* or Sert-p11cKO subjected to the emotion discrimination test (EDT). (**a**) There were no significant differences found in time sniffing of saline- or ketamine-treated Sert-p11cKO mice subjected to the EDT in a neutral *versus* neutral condition. (**b**) All groups presented a comparable discrimination index, regardless of stress stimulus. Values are expressed as means ± S.E.M (n = 8). Individual data are represented as dots (two-way ANOVA).

**Supplementary Table 1.** Description of statistical data of the Student’s t-test

| ***Parameter*** | ***t (df)*** | ***P value*** | ***Figure*** |
| --- | --- | --- | --- |
| *Tryptophan* | t _(10)_ = 0.83 | P=0.42 | Fig 3i,j |
| *5HTP* | t _(10)_ = 0.42 | P=0.68 | Fig 3i,j |
| *5HT* | t _(10)_ = 2.58 | P<0.05 | Fig 3i,j |
| *5HIAL* | t _(10)_ = 0.39 | P=0.69 | Fig 3i,j |
| *5HIAA* | t _(10)_ = 1.23 | P=0.24 | Fig 3i,j |
| *5HTOL* | t _(10)_ = 4.61 | P<0.01 | Fig 3i,j |
| *6OHM* | t _(10)_ = 5.19 | P<0.01 | Fig 3i,j |
| *DA* | t _(10)_ = 2.60 | P<0.05 | Suppl. Fig 5b |
| *3MT* | t _(10)_ = 0.02 | P=0.98 | Suppl. Fig 5b |
| *DOPAL* | t _(10)_ = 1.14 | P=0.27 | Suppl. Fig 5b |
| *DOPAC* | t _(10)_ = 0.37 | P=0.71 | Suppl. Fig 5b |
| *HVA* | t _(10)_ = 3.43 | P<0.01 | Suppl. Fig 5b |
| *Tyramine* | t _(10)_ = 1.70 | P=0.11 | Suppl. Fig 5b |
| *NE* | t _(10)_ = 2.71 | P<0.05 | Suppl. Fig 5b |
| *EP* | t _(10)_ = 0.09 | P=0.92 | Suppl. Fig 5b |
| *DOPEG* | t _(10)_ = 2.42 | P<0.05 | Suppl. Fig 5b |
| *MOPEG* | t _(10)_ = 4.88 | P<0.01 | Suppl. Fig 5b |
| *GABA* | t _(10)_ = 2.65 | P<0.05 | Suppl. Fig 5c |
| *GABA-H2O* | t _(10)_ = 2.74 | P<0.05 | Suppl. Fig 5c |
| *Spermidine* | t _(10)_ = 0.87 | P=0.39 | Suppl. Fig 5c |
| *Spermine* | t _(10)_ = 0.82 | P=0.43 | Suppl. Fig 5c |
| *Histidine* | t _(10)_ = 0.90 | P=0.38 | Suppl. Fig 5c |
| *Histamine* | t _(10)_ = 0.92 | P=0.37 | Suppl. Fig 5c |
| *Taurine* | t _(10)_ = 0.22 | P=0.82 | Suppl. Fig 5c |
| *Glycine* | t _(10)_ = 0.78 | P=0.78 | Suppl. Fig 5c |
| *Alanine* | t _(10)_ = 2.45 | P<0.05 | Suppl. Fig 5c |
| *Adenine* | t _(10)_ = 0.17 | P=0.86 | Suppl. Fig 5c |

5HTP - 5-hydroxytryptophan, 5HT - serotonin, 5HIAL - 5-hydroxyindoleacetaldehyde, 5HIAA - 5-hydroxyindoleacetic acid, 5-HTOL - 5-hydroxytryptophol, 6OHM -6-hydroxymelatonin, DA - dopamine, 3MT - 3-methoxytyramine, DOPAL - 3,4-Dihydroxyphenylacetaldehyde, DOPAC - 3,4-Dihydroxyphenylacetic acid, HVA - homovanillic acid, NE - norepinephrine, EP - epinephrine, DOPEG - dihydroxyphenylethylene glycol; MOPEG – methoxyhydroxyphenylglycol, GABA - gamma-aminobutyric acid, GABA-H2O - gamma-aminobutyric acid-H2O, S - stress, N – neutral.

| **Supplementary Table 2.** Description of statistical data of the two-way ANOVA | | | | | | | | | | | | |
| --- | --- | --- | --- | --- | --- | --- | --- | --- | --- | --- | --- | --- |
|  | **Group** | | | **Side** | | | **Group x Side** | | |  | | |
| ***Parameter*** | ***F (df)*** | | ***P value*** | ***F (df)*** | | ***P value*** | ***F (df)*** | | ***P value*** | ***Figure*** | | |
| *Ketamine: Time sniffing S vs N* | F _(3, 56)_ = 0.97 | | P=0.41 | F _(1, 56)_ = 36.4 | | P<0.01 | F _(3, 56)_ = 5.05 | | P<0.05 | Fig. 1c | | |
|  | **Stress** | | | **Treatment** | | | **Stress x Treatment** | | | | |  |
| ***Parameter*** | ***F (df)*** | | ***P value*** | ***F (df)*** | | ***P value*** | ***F (df)*** | | ***P value*** | ***Figure*** | | |
| *Ketamine: Discrimination index S vs N* | F _(1, 28)_ = 9.12 | | P<0.01 | F _(1, 28)_ = 4.26 | | P<0.05 | F _(1, 28)_ = 8.31 | | P<0.01 | Fig. 1d | | |
| *Ketamine: Sucrose preference* | F _(1, 28)_ = 9.15 | | P<0.01 | F _(1, 28)_ = 2.20 | | P=0.14 | F _(1, 28)_ = 6.40 | | P<0.05 | Fig. 1e | | |
| *Ketamine: Immobility time* | F _(1, 28)_ = 18.6 | | P<0.01 | F _(1, 28)_ = 8.97 | | P<0.01 | F _(1, 28)_ = 7.27 | | P<0.01 | Fig. 1f | | |
| *Ketamine: Distance traveled* | F _(1, 28)_ = 0.12 | | P=0.72 | F _(1, 28)_ = 0.05 | | P=0.82 | F _(1, 28)_ = 0.04 | | P=0.84 | Fig. 1g | | |
| *Tryptophan* | F _(1, 20)_ = 1.14 | | P=0.29 | F _(1, 20)_ = 0.18 | | P=0.67 | F _(1, 20)_ = 0.21 | | P=0.65 | Fig. 1l,m | | |
| *5HTP* | F _(1, 20)_ = 1.14 | | P=0.32 | F _(1, 20)_ = 1.05 | | P=0.31 | F _(1, 20)_ = 0.01 | | P=0.89 | Fig. 1l,n | | |
| *5HT* | F _(1, 20)_ = 9.89 | | P<0.01 | F _(1, 20)_ = 4.22 | | P=0.053 | F _(1, 20)_ = 4.99 | | P<0.05 | Fig. 2l,o | | |
| *5HIAL* | F _(1, 20)_ = 0.01 | | P=0.93 | F _(1, 20)_ = 0.29 | | P=0.59 | F _(1, 20)_ = 0.14 | | P=0.71 | Fig. 1p,q | | |
| *5HIAA* | F _(1, 20)_ = 0.49 | | P=0.48 | F _(1, 20)_ = 1.09 | | P=0.30 | F _(1, 20)_ = 0.30 | | P=0.58 | Fig. 1p,r | | |
| *5HTOL* | F _(1, 20)_ = 7.52 | | P<0.01 | F _(1, 20)_ = 1.68 | | P=0.20 | F _(1, 20)_ = 7.57 | | P<0.01 | Fig. 1p,s | | |
|  | **Group** | | | **Side** | | | **Group x Side** | | |  | | |
| ***Parameter*** | ***F (df)*** | | ***P value*** | ***F (df)*** | | ***P value*** | ***F (df)*** | | ***P value*** | ***Figure*** | | |
| *Ketamine: Time sniffing N vs N* | F _(3, 56)_ = 1.82 | | P=0.15 | F _(1, 56)_ = 0.81 | | P=0.37 | F _(3, 56)_ = 0.73 | | P=0.53 | Suppl. Fig. 1a | | |
|  | **Stress** | | | **Treatment** | | | **Stress x Treatment** | | |  | | |
| ***Parameter*** | ***F (df)*** | | ***P value*** | ***F (df)*** | | ***P value*** | ***F (df)*** | | ***P value*** | ***Figure*** | | |
| *Ketamine: Discrimination index N vs N* | F _(3, 56)_ = 0.07 | | P=0.78 | F _(1, 56)_ = 0.25 | | P=0.61 | F _(3, 56)_ = 3.46 | | P=0.07 | Suppl. Fig. 1b | | |
| *DA* | F _(1, 20)_ = 4.37 | | P<0.05 | F _(1, 20)_ = 1.33 | | P=0.26 | F _(1, 20)_ = 2.28 | | P=0.14 | Suppl. Fig. 2b | | |
| *3MT* | F _(1, 20)_ = 1.82 | | P=0.19 | F _(1, 20)_ = 4.67 | | P<0.05 | F _(1, 20)_ = 0.40 | | P=0.53 | Suppl. Fig. 2c | | |
| *DOPAL* | F _(1, 20)_ = 20.4 | | P<0.01 | F _(1, 20)_ = 0.08 | | P=0.77 | F _(1, 20)_ = 0.10 | | P=0.74 | Suppl. Fig. 2d | | |
| *DOPAC* | F _(1, 20)_ = 0.38 | | P=0.54 | F _(1, 20)_ = 0.14 | | P=0.70 | F _(1, 20)_ = 0.72 | | P=0.40 | Suppl. Fig. 2e | | |
| *HVA* | F _(1, 20)_ = 7.04 | | P<0.05 | F _(1, 20)_ = 0.24 | | P=0.62 | F _(1, 20)_ = 0.44 | | P=0.51 | Suppl. Fig. 2f | | |
| *Tyramine* | F _(1, 20)_ = 0.14 | | P=0.70 | F _(1, 20)_ = 0.06 | | P=0.79 | F _(1, 20)_ = 0.26 | | P=0.60 | Suppl. Fig. 2g | | |
| *NE* | F _(1, 20)_ = 0.55 | | P=0.46 | F _(1, 20)_ = 0.01 | | P=0.89 | F _(1, 20)_ = 1.71 | | P=0.20 | Suppl. Fig. 2h | | |
| *EP* | F _(1, 20)_ = 3.64 | | P=0.07 | F _(1, 20)_ = 5.47 | | P<0.05 | F _(1, 20)_ = 0.43 | | P=0.51 | Suppl. Fig. 2i | | |
| *DOPEG* | F _(1, 20)_ = 2.17 | | P=0.15 | F _(1, 20)_ = 0.86 | | P=0.36 | F _(1, 20)_ = 0.01 | | P=0.98 | Suppl. Fig. 2j | | |
| *MOPEG* | F _(1, 20)_ = 8.73 | | P<0.01 | F _(1, 20)_ = 0.22 | | P=0.64 | F _(1, 20)_ = 0.02 | | P=0.93 | Suppl. Fig. 2k | | |
| *GABA* | F _(1, 20)_ = 7.01 | | P<0.05 | F _(1, 20)_ = 0.37 | | P=0.54 | F _(1, 20)_ = 0.39 | | P=0.53 | Suppl. Fig. 2l | | |
| *GABA-H2O* | F _(1, 20)_ = 22.1 | | P<0.01 | F _(1, 20)_ = 0.95 | | P=0.34 | F _(1, 20)_ = 1.80 | | P=0.19 | Suppl. Fig. 2m | | |
| *Spermidine* | F _(1, 20)_ = 0.03 | | P=0.90 | F _(1, 20)_ = 0.05 | | P=0.81 | F _(1, 20)_ = 0.01 | | P=0.99 | Suppl. Fig. 2n | | |
| *Spermine* | F _(1, 20)_ = 0.52 | | P=0.47 | F _(1, 20)_ = 0.26 | | P=0.61 | F _(1, 20)_ = 0.94 | | P=0.34 | Suppl. Fig. 2o | | |
| *Histidine* | F _(1, 20)_ = 3.57 | | P=0.07 | F _(1, 20)_ = 0.04 | | P=0.84 | F _(1, 20)_ = 1.99 | | P=0.17 | Suppl. Fig. 2p | | |
| *Histamine* | F _(1, 20)_ = 2.23 | | P=0.15 | F _(1, 20)_ = 2.05 | | P=0.16 | F _(1, 20)_ = 0.04 | | P=0.89 | Suppl. Fig. 2q | | |
| *Taurine* | F _(1, 20)_ = 3.73 | | P=0.06 | F _(1, 20)_ = 0.81 | | P=0.37 | F _(1, 20)_ = 0.08 | | P=0.77 | Suppl. Fig. 2r | | |
| *Glycine* | F _(1, 20)_ = 6.70 | | P<0.05 | F _(1, 20)_ = 0.12 | | P=0.73 | F _(1, 20)_ = 0.92 | | P=0.34 | Suppl. Fig. 2s | | |
| *Alanine* | F _(1, 20)_ = 4.74 | | P<0.05 | F _(1, 20)_ = 0.16 | | P=0.68 | F _(1, 20)_ = 0.58 | | P=0.43 | Suppl. Fig. 2t | | |
| *Adenine* | F _(1, 20)_ = 6.24 | | P<0.05 | F _(1, 20)_ = 1.38 | | P=0.25 | F _(1, 20)_ = 0.56 | | P=0.46 | Suppl. Fig. 2u | | |
| *6OHM* | F _(1, 20)_ = 10.6 | | P<0.01 | F _(1, 20)_ = 2.78 | | P=0.11 | F _(1, 20)_ = 0.56 | | P=0.46 | Suppl. Fig. 2v | | |
|  | **Stress** | | | **Treatment** | | | **Stress x Treatment** | | | |  | |
| ***Parameter*** | ***F (df)*** | | ***P value*** | ***F (df)*** | | ***P value*** | ***F (df)*** | | ***P value*** | ***Figure*** | | |
| *Ketamine: p11 mRNA FI/DRN* | F _(1, 20)_ = 25.7 | | P<0.01 | F _(1, 20)_ = 7.33 | | P<0.01 | F _(1, 20)_ = 4.16 | | P=0.054 | Fig. 2a,b | | |
| *Ketamine: p11 mRNA FI/cell* | F _(1, 796)_ = 90.8 | | P<0.01 | F _(1, 796)_ = 138.1 | | P<0.01 | F _(1, 796)_ = 9.79 | | P<0.01 | Fig. 2c,d | | |
|  | **Group** | | | **Side** | | | **Group x Side** | | |  | | |
| ***Parameter*** | ***F (df)*** | | ***P value*** | ***F (df)*** | | ***P value*** | ***F (df)*** | | ***P value*** | ***Figure*** | | |
| *P11HET: Time sniffing S vs N* | F _(3, 56)_ = 7.11 | | P<0.01 | F _(1, 56)_ = 53.1 | | P=0.14 | F _(3, 56)_ = 5.43 | | P<0.01 | Fig. 3c | | |
|  | **Stress** | | | **Genotype** | | | **Stress x Genotype** | | |  | | |
| ***Parameter*** | ***F (df)*** | | ***P value*** | ***F (df)*** | | ***P value*** | ***F (df)*** | | ***P value*** | ***Figure*** | | |
| *P11HET: Discrimination index S vs N* | F _(1, 28)_ = 2.77 | | P=0.10 | F _(1, 28)_ = 7.24 | | P<0.05 | F _(1, 28)_ = 2.50 | | P=0.16 | Fig. 3d | | |
| *P11HET: Sucrose preference* | F _(1, 28)_ = 12.5 | | P<0.01 | F _(1, 28)_ = 13.1 | | P<0.01 | F _(1, 28)_ = 5.61 | | P<0.05 | Fig. 3e | | |
| *P11HET: Immobility time* | F _(1, 28)_ = 7.74 | | P<0.01 | F _(1, 28)_ = 8.22 | | P<0.01 | F _(1, 28)_ = 19.4 | | P<0.01 | Fig. 3f | | |
| *P11HET: Distance traveled* | F _(1, 28)_ = 0.64 | | P=0.43 | F _(1, 28)_ = 0.69 | | P=0.41 | F _(1, 28)_ = 0.18 | | P=0.67 | Fig. 3g | | |
|  | **Group** | | | **Side** | | | **Group x Side** | | |  | | |
| ***Parameter*** | ***F (df)*** | | ***P value*** | ***F (df)*** | | ***P value*** | ***F (df)*** | | ***P value*** | ***Figure*** | | |
| *P11KO: Time sniffing S vs N* | F _(3, 56)_ = 4.18 | | P<0.05 | F _(1, 56)_ = 18.4 | | P<0.01 | F _(3, 56)_ = 14.7 | | P<0.01 | Fig. 3j | | |
|  | **Stress** | | | **Genotype** | | | **Stress x Genotype** | | |  | | |
| ***Parameter*** | ***F (df)*** | | ***P value*** | ***F (df)*** | | ***P value*** | ***F (df)*** | | ***P value*** | ***Figure*** | | |
| *P11KO: Discrimination index S vs N* | F _(1, 28)_ = 3.95 | | P=0.056 | F _(1, 28)_ = 45.3 | | P<0.01 | F _(1, 28)_ = 1.80 | | P=0.18 | Fig. 3k | | |
| *P11KO: Sucrose preference* | F _(1, 28)_ = 1.25 | | P=0.27 | F _(1, 28)_ = 38.9 | | P<0.01 | F _(1, 28)_ = 1.92 | | P=0.17 | Fig. 3l | | |
| *P11KO: Immobility time* | F _(1, 28)_ = 4.86 | | P<0.05 | F _(1, 28)_ = 89.9 | | P<0.01 | F _(1, 28)_ = 7.27 | | P<0.05 | Fig. 3m | | |
| *P11KO: Distance traveled* | F _(1, 28)_ = 0.03 | | P=0.98 | F _(1, 28)_ = 0.05 | | P=0.94 | F _(1, 28)_ = 0.07 | | P=0.79 | Fig. 3n | | |
|  | **Group** | | | **Side** | | | **Group x Side** | | |  | | |
| ***Parameter*** | ***F (df)*** | | ***P value*** | ***F (df)*** | | ***P value*** | ***F (df)*** | | ***P value*** | ***Figure*** | | |
| *P11HET: Time sniffing N vs N* | F _(3, 56)_ = 1.91 | | P=0.13 | F _(1, 56)_ = 1.35 | | P=0.24 | F _(3, 56)_ = 0.13 | | P=0.35 | Suppl. Fig. 3a | | |
| *P11KO: Time sniffing N vs N* | F _(3, 56)_ = 1.82 | | P=0.15 | F _(1, 56)_ = 0.32 | | P=0.57 | F _(3, 56)_ = 0.57 | | P=0.63 | Suppl. Fig. 3c | | |
|  | **Stress** | | | **Genotype** | | | **Stress x Genotype** | | |  | | |
| ***Parameter*** | ***F (df)*** | | ***P value*** | ***F (df)*** | | ***P value*** | ***F (df)*** | | ***P value*** | ***Figure*** | | |
| *P11HET: Discrimination index N vs N* | F _(1, 28)_ = 0.26 | | P=0.61 | F _(1, 28)_ = 1.38 | | P=0.25 | F _(1, 28)_ = 0.41 | | P=0.52 | Suppl. Fig. 3b | | |
| *P11KO: Discrimination index N vs N* | F _(1, 28)_ = 0.27 | | P=0.60 | F _(1, 28)_ = 0.08 | | P=0.98 | F _(1, 28)_ = 0.93 | | P=0.34 | Suppl. Fig. 3d | | |
|  | **Group** | | | **Side** | | | **Group x Side** | | |  | | |
| ***Parameter*** | ***F (df)*** | | ***P value*** | ***F (df)*** | | ***P value*** | ***F (df)*** | | ***P value*** | ***Figure*** | | |
| *SERTp11cKO: Time sniffing S vs N* | F _(3, 56)_ = 11.78 | | P<0.01 | F _(1, 56)_ = 20.8 | | P<0.01 | F _(3, 56)_ = 5.21 | | P<0.01 | Fig. 4d | | |
|  | **Stress** | | | **Genotype** | | | **Stress x Genotype** | | |  | | |
| ***Parameter*** | ***F (df)*** | | ***P value*** | ***F (df)*** | | ***P value*** | ***F (df)*** | | ***P value*** | ***Figure*** | | |
| *SERTp11cKO: Discrimination index S vs N* | F _(1, 28)_ = 0.05 | | P=0.82 | F _(1, 28)_ = 6.12 | | P<0.05 | F _(1, 28)_ = 0.24 | | P=0.62 | Fig. 4e | | |
| *SERTp11cKO: Sucrose preference* | F _(1, 28)_ = 9.47 | | P<0.01 | F _(1, 28)_ = 8.49 | | P<0.01 | F _(1, 28)_ = 6.41 | | P<0.05 | Fig. 4f | | |
| *SERTp11cKO: Immobility time* | F _(1, 28)_ = 15.4 | | P<0.01 | F _(1, 28)_ = 17.8 | | P<0.01 | F _(1, 28)_ = 21.37 | | P<0.01 | Fig. 4g | | |
| *SERTp11cKO: Distance traveled* | F _(1, 28)_ = 0.38 | | P=0.54 | F _(1, 28)_ = 0.17 | | P=0.67 | F _(1, 28)_ = 0.28 | | P=0.59 | Fig. 4h | | |
|  | **Brain region** | | | **Genotype** | | | **Brain Region x Genotype** | | |  | | |
|  | ***F (df)*** | ***P value*** | | ***F (df)*** | ***P value*** | | ***F (df)*** | ***P value*** | | ***Figure*** | | |
| *SERTp11cKO: virus injection* | F _(3, 16)_ = 13.2 | P<0.01 | | F _(1, 16)_ = 29.4 | P<0.01 | | F _(3, 15)_ = 13.3 | P<0.01 | | Suppl. Fig. 4b | | |
|  | **Group** | | | **Side** | | | **Group x Side** | | |  | | |
| ***Parameter*** | ***F (df)*** | | ***P value*** | ***F (df)*** | | ***P value*** | ***F (df)*** | | ***P value*** | ***Figure*** | | |
| *SERTp11cKO: Time sniffing N vs N* | F _(3, 56)_ = 0.91 | | P=0.43 | F _(1, 56)_ = 0.16 | | P=0.68 | F _(3, 56)_ = 0.14 | | P=0.93 | Suppl. Fig. 4c | | |
|  | **Stress** | | | **Genotype** | | | **Stress x Genotype** | | |  | | |
| ***Parameter*** | ***F (df)*** | | ***P value*** | ***F (df)*** | | ***P value*** | ***F (df)*** | | ***P value*** | ***Figure*** | | |
| *SERTp11cKO: Discrimination index N vs N* | F _(1, 28)_ = 0.67 | | P=0.41 | F _(1, 28)_ = 0.51 | | P=0.48 | F _(1, 28)_ = 0.36 | | P=0.54 | Suppl. Fig. 4d | | |
|  | **Group** | | | **Side** | | | **Group x Side** | | |  | | |
| ***Parameter*** | ***F (df)*** | | ***P value*** | ***F (df)*** | | ***P value*** | ***F (df)*** | | ***P value*** | ***Figure*** | | |
| *ChATp11cKO: Time sniffing S vs N* | F _(3, 56)_ = 5.94 | | P<0.01 | F _(1, 56)_ = 1.84 | | P=0.17 | F _(3, 56)_ = 11.8 | | P<0.01 | Suppl. Fig. 6d | | |
| *ChATp11cKO: Time sniffing N vs N* | F _(3, 56)_ = 0.86 | | P=0.46 | F _(1, 56)_ = 1.78 | | P=0.18 | F _(3, 56)_ = 0.93 | | P=0.42 | Suppl. Fig. 6f | | |
|  | **Stress** | | | **Genotype** | | | **Stress x Genotype** | | |  | | |
| ***Parameter*** | ***F (df)*** | | ***P value*** | ***F (df)*** | | ***P value*** | ***F (df)*** | | ***P value*** | ***Figure*** | | |
| *ChATp11cKO: Discrimination index S vs N* | F _(1, 28)_ = 0.27 | | P=0.60 | F _(1, 28)_ = 28.1 | | P<0.01 | F _(1, 28)_ = 0.78 | | P=0.38 | Suppl. Fig. 6e | | |
| *ChATp11cKO: Discrimination index N vs N* | F _(1, 28)_ = 1.07 | | P=0.31 | F _(1, 28)_ = 2.41 | | P=0.13 | F _(1, 28)_ = 0.57 | | P=0.45 | Suppl. Fig. 6g | | |
| *ChATp11cKO: Sucrose preference* | F _(1, 28)_ = 0.37 | | P=0.54 | F _(1, 28)_ = 34.2 | | P<0.01 | F _(1, 28)_ = 0.09 | | P=0.92 | Suppl. Fig. 6h | | |
| *ChATp11cKO: Immobility time* | F _(1, 28)_ = 0.09 | | P=0.75 | F _(1, 28)_ = 40.2 | | P<0.01 | F _(1, 28)_ = 0.99 | | P=0.32 | Suppl. Fig. 6i | | |
| *ChATp11cKO: Distance traveled* | F _(1, 28)_ = 0.75 | | P=0.39 | F _(1, 28)_ = 0.59 | | P=0.44 | F _(1, 28)_ = 0.03 | | P=0.84 | Suppl. Fig. 6j | | |
|  | **Group** | | | **Side** | | | **Group x Side** | | |  | | |
| ***Parameter*** | ***F (df)*** | | ***P value*** | ***F (df)*** | | ***P value*** | ***F (df)*** | | ***P value*** | ***Figure*** | | |
| *AAV-Cre: Time sniffing S vs N* | F _(3, 56)_ = 1.25 | | P=0.29 | F _(1, 56)_ = 32.3 | | P<0.01 | F _(3, 56)_ = 8.95 | | P<0.01 | Fig. 5d | | |
|  | **Stress** | | | **AAV Injection** | | | **Stress x Genotype** | | |  | | |
| ***Parameter*** | ***F (df)*** | | ***P value*** | ***F (df)*** | | ***P value*** | ***F (df)*** | | ***P value*** | ***Figure*** | | |
| *AAV-Cre: Discrimination index S vs N* | F _(1, 28)_ = 3.22 | | P=0.08 | F _(1, 28)_ = 28.6 | | P<0.01 | F _(1, 28)_ = 0.02 | | P=0.87 | Fig. 5e | | |
| *AAV-Cre: Sucrose preference* | F _(1, 28)_ = 4.82 | | P<0.05 | F _(1, 28)_ = 14.7 | | P<0.01 | F _(1, 28)_ = 18.6 | | P<0.01 | Fig. 5f | | |
| *AAV-Cre: Immobility time* | F _(1, 28)_ = 2.90 | | P=0.09 | F _(1, 28)_ = 8.59 | | P<0.01 | F _(1, 28)_ = 4.22 | | P<0.05 | Fig. 5g | | |
| *AAV-Cre: Distance traveled* | F _(1, 28)_ = 0.64 | | P=0.42 | F _(1, 28)_ = 0.42 | | P=0.52 | F _(1, 28)_ = 0.21 | | P=0.64 | Fig. 5h | | |
| *p11 levels* | F _(1, 20)_ = 0.13 | | P=0.71 | F _(1, 20)_ = 18.76 | | P<0.01 | F _(1, 20)_ = 0.10 | | P=0.75 | Suppl. Fig. 7a | | |
|  | **Group** | | | **Side** | | | **Group x Side** | |  |  | | |
| ***Parameter*** | ***F (df)*** | | ***P value*** | ***F (df)*** | | ***P value*** | ***F (df)*** | | ***P value*** | ***Figure*** | | |
| *AAV-Cre: Time sniffing N vs N* | F _(3, 56)_ = 0.91 | | P=0.43 | F _(1, 56)_ = 0.16 | | P=0.68 | F _(3, 56)_ = 0.14 | | P=0.14 | Suppl. Fig. 7c | | |
|  | **Stress** | | | **Genotype** | | | **Stress x Genotype** | | |  | | |
| ***Parameter*** | ***F (df)*** | | ***P value*** | ***F (df)*** | | ***P value*** | ***F (df)*** | | ***P value*** | ***Figure*** | | |
| *AAV-Cre: Discrimination index N vs N* | F _(3, 56)_ = 0.67 | | P=0.41 | F _(1, 56)_ = 0.51 | | P=0.48 | F _(3, 56)_ = 0.36 | | P=0.54 | Suppl. Fig. 7d | | |
|  | **Group** | | | **Side** | | | **Group x Side** | | |  | | |
| ***Parameter*** | ***F (df)*** | | ***P value*** | ***F (df)*** | | ***P value*** | ***F (df)*** | | ***P value*** | ***Figure*** | | |
| *Ketamine Sertp11cKO: Time sniffing S vs N* | F _(7, 112)_ = 9.78 | | P<0.01 | F _(1, 112)_ = 23.1 | | P<0.01 | F _(7, 112)_ = 4.57 | | P<0.01 | Fig. 6c | | |
|  | **Stress** | | | **Treatment** | | | **Stress x Treatment** | | |  | | |
| ***Parameter*** | ***F (df)*** | | ***P value*** | ***F (df)*** | | ***P value*** | ***F (df)*** | | ***P value*** | ***Figure*** | | |
| *Ket Sertp11cKO: Discrimination index S vs N* | F _(3, 56)_ = 14.8 | | P<0.01 | F _(1, 56)_ = 6.21 | | P<0.05 | F _(3, 56)_ = 3.66 | | P<0.05 | Fig. 6d | | |
| *Ketamine Sertp11cKO: Sucrose preference* | F _(3, 56)_ = 45.5 | | P<0.01 | F _(1, 56)_ = 5.14 | | P<0.01 | F _(3, 56)_ = 4.86 | | P<0.01 | Fig. 6e | | |
| *Ketamine Sertp11cKO: Immobility time* | F _(3, 56)_ = 78.1 | | P<0.01 | F _(1, 56)_ = 6.28 | | P<0.01 | F _(3, 56)_ = 3.59 | | P<0.05 | Fig. 6f | | |
| *Ketamine Sertp11cKO: Distance traveled* | F _(3, 56)_ = 0.08 | | P=0.96 | F _(1, 56)_ = 0.07 | | P=0.78 | F _(3, 56)_ = 0.11 | | P=0.95 | Fig. 6g | | |
|  | **Group** | | | **Side** | | | **Group x Side** | | |  | | |
| ***Parameter*** | ***F (df)*** | | ***P value*** | ***F (df)*** | | ***P value*** | ***F (df)*** | | ***P value*** | ***Figure*** | | |
| *Ketamine Sertp11cKO: Time sniffing N vs N* | F _(7, 112)_ = 0.91 | | P=0.50 | F _(1, 112)_ = 0.46 | | P=0.49 | F _(7, 112)_ = 0.12 | | P=0.99 | Suppl. Fig. 8a | | |
|  | **Stress** | | | **Treatment** | | | **Stress x Treatment** | | |  | | |
| ***Parameter*** | ***F (df)*** | | ***P value*** | ***F (df)*** | | ***P value*** | ***F (df)*** | | ***P value*** | ***Figure*** | | |
| *Ket Sertp11cKO: Discrimination index N vs N* | F _(7, 112)_ = 0.06 | | P=0.81 | F _(1, 112)_ = 0.28 | | P=0.83 | F _(7, 112)_ = 0.71 | | P=0.54 | Suppl. Fig. 8b | | |

5HTP - 5-hydroxytryptophan, 5HT - serotonin, 5HIAL - 5-hydroxyindoleacetaldehyde, 5HIAA - 5-hydroxyindoleacetic acid, 5-HTOL - 5-hydroxytryptophol, 6OHM -6-hydroxymelatonin, DA - dopamine, 3MT - 3-methoxytyramine, DOPAL - 3,4-Dihydroxyphenylacetaldehyde, DOPAC - 3,4-Dihydroxyphenylacetic acid, HVA - homovanillic acid, NE - norepinephrine, EP - epinephrine, DOPEG - dihydroxyphenylethylene glycol; MOPEG – methoxyhydroxyphenylglycol, GABA - gamma-aminobutyric acid, GABA-H2O - gamma-aminobutyric acid-H2O, S - stress, N – neutral, KO - knockout, WT, wild-type, HET - heterozygous.
